# An Evolutionary Analysis of B-Box Transcription Factors in Strawberry Reveals the Role of FaBBx28c1 in the Regulation of Flowering Time

**DOI:** 10.3390/ijms222111766

**Published:** 2021-10-29

**Authors:** Yuntian Ye, Yongqiang Liu, Xiaolong Li, Guangyi Wang, Quan Zhou, Qing Chen, Jiale Li, Xiaorong Wang, Haoru Tang

**Affiliations:** 1 College of Horticulture, Sichuan Agricultural University, Chengdu 611130, China; yeyuntian@sicau.cn (Y.Y.); liuyongqiang_95@163.com (Y.L.); lixiaolong_96@163.com (X.L.); Wanggy0315@163.com (G.W.); supnovel@gmail.com (Q.C.); lijiale815@163.com (J.L.); wangxr@sicau.edu.cn (X.W.); 2 Viikki Plant Science Centre, Department of Agricultural Sciences, University of Helsinki, 00790 Helsinki, Finland; quan.zhou@helsinki.fi; 3 Institute of Pomology and Olericulture, Sichuan Agricultural University, Chengdu 611130, China

**Keywords:** strawberry, B-box protein, gene family, flowering time, gene expression profiling

## Abstract

Flowering connects vegetative and generative developmental phases and plays a significant role in strawberry production. The mechanisms that regulate strawberry flowering time are unclear. B-box transcription factors (BBXs) play important roles in the flowering time regulation of plants. Nevertheless, BBXs in octoploid cultivated strawberry (*Fragaria ananassa*) and their functions in flowering time regulation have not been identified. Here, we identified 51 *FaBBXs* from cultivated strawberry and 16 *FvBBXs* from diploid wild strawberry (*Fragaria vesca*), which were classified into five groups according to phylogenetic analysis. Further evolutionary analysis showed that whole-genome duplication or segmental duplication is a crucial factor that leads to the expansion of the BBX gene family in two strawberry species. Moreover, some loss and acquisition events of *FaBBX* genes were identified in the genome of cultivated strawberry that could have affected traits of agronomic interest, such as fruit quality. The promoters of *FaBBX* genes showed an enrichment in light-responsive, *cis*-regulatory elements, with 16 of these genes showing changes in their transcriptional activity in response to blue light treatment. On the other hand, *FaBBX28c1*, whose transcriptional activity is reduced in response to blue light, showed a delay in flowering time in *Arabidopsis* transgenic lines, suggesting its role in the regulation of flowering time in cultivated strawberry. Our results provide new evolutionary insight into the BBX gene family in cultivated strawberry and clues regarding their function in flowering time regulation in plants.

## 1. Introduction

Transcription factors play key roles in various plant biological processes, such as stress responses, development regulation, and secondary metabolic pathway mediation. A genome-wide identification of transcription factors in Arabidopsis shows that a large class of 1500 transcription factors is encoded by the genome, approximately 45% of which are specific to plants [1]. Among them, so-called B-box proteins (BBXs) are a class of zinc finger transcription factors that contain one or two B-box conserved domains mediating protein–protein interactions [2]. Systematic identification of the BBX family in *Arabidopsis* has identified 32 AtBBXs (AtBBX1-AtBBX32) that can be further classified into 5 groups (Group I–Group V) on the basis of the presence of domains [3]. The members of Group I contain two B-box domains in tandem and a CCT (CONSTANS, CO-like, and TOC1) domain. Structure Group II members are similar to Group I, both of which contain two tandem B-box domains and one CCT domain; however, differences in their second B-box domains were observed. The AtBBX members of structure Group III contain a single B-box domain in association with a CCT domain. Structure Group IV contains two B-boxes, and no CCT domain is found. Finally, structure Group V consists of members with only a single B-box domain [2,3].

Numerous analyses concerning the biological functions of *AtBBXs* have been reported. The biological functions of *AtBBXs* are associated with multiple physiological and biochemical processes, including developmental regulation, flowering time regulation, and response to external stress [2,4]. AtBBX28 is a negative regulator of AtHY5 that participates in the COP1-HY5 pathway mediating photomorphogenic development via a physical interaction with the AtHY5 protein, which represses the activity of AtHY5 in a dose-dependent manner in *Arabidopsis* [5]. In addition, AtBBX28 can interact with AtCO (CONSTANS), which is a transcriptional activator of *AtFT* (Flowering locus T) in flowering time regulation pathway. Overexpression of *AtBBX28* leads a late flowering phenotype with decreased *AtFT* expression, which indicates that AtBBX28 protein is a negative regulator of flowering time in *Arabidopsis* [6]. The impact of low ambient temperature on the function of *AtBBX28* and *AtBBX29* has been revealed. The transcript levels of both *AtBBX28* and *AtBBX29* are induced by low-temperature treatment. However, under the low temperature condition of 16 °C, the double mutant of *AtBBX28* and *AtBBX29* shows a late flowering time accompanied by decreasing expression levels of *AtFT* and *AtCO*. In contrast, these phenotypes are not observed at 29 °C [7].

In addition, recent studies have expanded insights into the function of BBX proteins in non-model plant physiological processes, including tolerance to stressful environments [8,9], flowering time regulation [8,10,11,12], and biosynthesis of secondary metabolites [12,13,14]. Cultivated strawberry is an important fruit crop that is globally cultivated with high economic value. The genomic data of the allo-octoploid strawberry (2n = 8x = 56) support the hypothesis that octoploid strawberry originated through successive stages of polyploidization involving four genitor species: *Fragaria nippoinca*, *Fragaria innumea*, *Fragaria viridis*, and *Fragaria vesca*. Among them, the *F. vesca*-like subgenome was found to be the single dominant subgenome [15]. The BBX transcription factor family has been identified in diploid wild strawberry (*Fragaria vesca*) [16]. However, little is known about the BBX family in cultivated strawberry and the evolutionary relationship between *FaBBXs* and *FvBBXs*.

Extending the cropping season, which can be achieved by breeding for the cultivars that flower perpetually throughout the growing season, is one aim in strawberry breeding programs. The understanding of the genetic mechanisms controlling flower induction in strawberry could help breeders in developing new cultivars with the desired flowering characteristics [17]. Moreover, the blue light could promote flowering in both wild strawberry and cultivated strawberry [18,19]. Our previous transcriptome analysis of the accelerated flowering time of cultivated strawberry under blue light quality treatments further showed that the DEGs (differentially expressed genes) could be significantly enriched in BBX gene family [19]. To date, FvCO is the only BBX that has been identified as a functional regulator of flowering time in wild strawberry [10]. More understanding about the role of other BBXs from strawberry in the regulation of flowering time is still lacking, and it necessitates further investigation.

In the present study, the BBX family members in cultivated strawberry and wild strawberry were systematically identified on the basis of genome data. Then, the evolutionary relationship between *FaBBXs* and *FvBBXs* was explored. Finally, we characterized one member, *FaBBX28c1*, by ectopic expression. Our results provide information on the evolution of BBXs in the two aforementioned strawberry species and new insight into the potential biological functions of BBX proteins concerning the regulation of flowering time in strawberry.

## 2. Results

### 2.1. B-Box Genes in Wild Strawberry and Cultivated Strawberry

**Figure 1:**
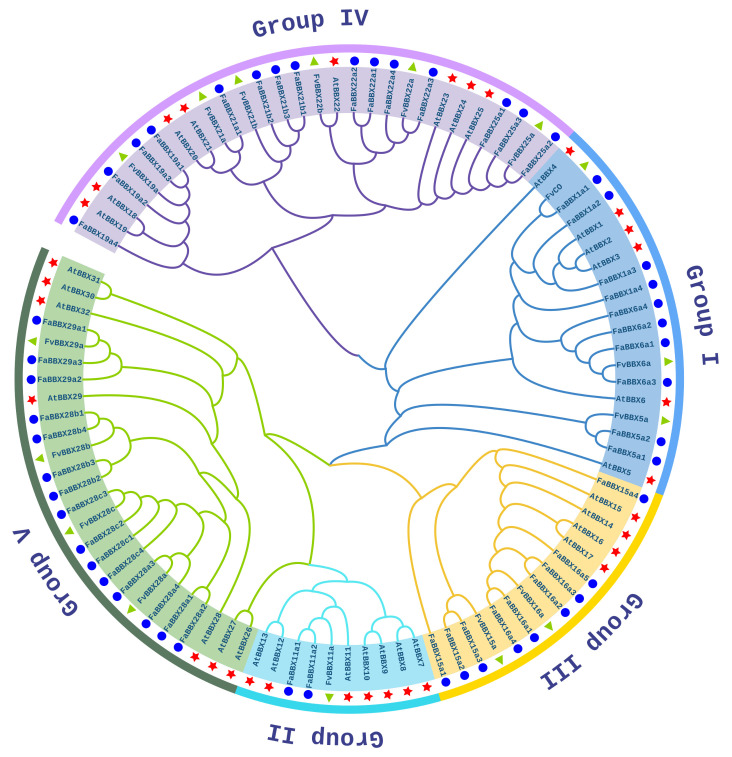
An unrooted phylogenetic tree of BBX proteins from *Arabidopsis* and two strawberry species. The BBX proteins from different species are marked with different shapes, including red stars (BBX proteins from *Arabidopsis*), blue circles (BBX proteins from cultivated strawberry), and green triangles (BBX proteins from wild strawberry).

We identified 16 *FvBBX* genes from wild strawberry and 51 *FaBBX* genes from the cultivated strawberry genome (Table S1). The names of BBXs were assigned on the basis of the phylogenetic tree (Figure 1, Table S1).

FvCO (gene04172) has been reported as a regulator of flowering time in a previous report [10]. An alignment showed an identity of mRNA sequences between FvCO and FvBBX1 (Figure S1). Therefore, we used FvCO instead of FvBBX1 in our subsequent analyses.

The physical and chemical properties of BBX proteins in strawberry showed diverse peptide lengths, molecular weights, and isoelectric points (Table S2, Figure S2). The peptide length of BBX proteins in strawberry ranges from 70 (FvBBX22b) to 485 (FaBBX16a4). The isoelectric points of BBX proteins range from 3.94 (FaBBX28a2) to 8.65 (FvBBX29a). The molecular weight parameters of BBX proteins range from 7667.8 Da (FvBBX22b) to 54,135.4 Da (FaBBX16a4).

### 2.2. Phylogenetic Analysis

The evolutionary relationships of BBX proteins among wild strawberry (FvBBXs), cultivated strawberry (FaBBXs), and *Arabidopsis* (AtBBXs) were inferred using a maximum likelihood phylogenetic analysis. According to the topology of the phylogenetic tree and a previous report in *Arabidopsis* [2,3], BBX proteins can be divided into five groups (designated Groups I–V) (Figure 1). All five groups contain BBX proteins from *Arabidopsis* and two strawberry species, which suggests a common ancient origin of BBX proteins from these species. Group I contains 3 FvBBXs and 10 FaBBXs. Only one FvBBX (FvBBX11a) and two FaBBXs (FaBBX11a1 and FaBBX11a2) are classified into Group II. Group III contains two FvBBXs (FvBBX15a and FvBBX16a) and nine FaBBXs. In total, 6 FvBBXs and 15 FaBBXs are classified into Group IV, which is the largest group in BBX gene families in wild strawberry and cultivated strawberry. Group V contains 4 FvBBXs and 15 FaBBXs. Moreover, the clade, which comprises AtBBX28, two FvBBX28s, and eight FaBBX28s, implies an expansion after the speciation event between the strawberries’ ancestor species and *Arabidopsis*.

The number of *FaBBXs* from the octoploid cultivated strawberry is less than four times the number of *FvBBXs* from the diploid wild strawberry. To understand this numerical imbalance, we have presented the details of the phylogenetic clades containing BBX genes from wild strawberry and cultivated strawberry in Table 1. Eight clades contain the imbalanced number of *FvBBXs* and *FaBBXs*, namely, the clades *FvBBX5a-FaBBX5a2*, *FvBBX11a-FaBBX11a2*, *FvBBX16a-FaBBX16a5*, *FvBBX21a-FaBBX21a1*, *FvBBX21b-FaBBX21b3*, *FvBBX22b*, *FvBBX25-FaBBX25a3*, and *FvBBX29a-FaBBX29a3*. For most of these clades, such as *FvBBX5a-FaBBX5a2*, the *FaBBXs* are not found in all four subgenomes, which could be caused by the gene loss event during the evolution of the four progenitor wild strawberry and cultivated strawberry. On the contrary, for the clade *FvBBX16a-FaBBX16a5*, the gene duplication could lead one more BBX genes in the *F. vesca*-like subgenome. In the clade *FvBBX15a-FaBBX15a4*, two *FaBBXs* were identified in the *F. vesca*-like subgenome, while no *FaBBX* was found in the *F.niponica*-like subgenome. For this clade, the gene flow from the *F. niponica*-like subgenome to the *F. vesca*-like subgenome during meiosis, resulting an exchange of the fragment between subgenomes, could be an explanation, according to a previous report [16].

### 2.3. Gene Structure and Conserved Domain Analysis

**Figure 2:**
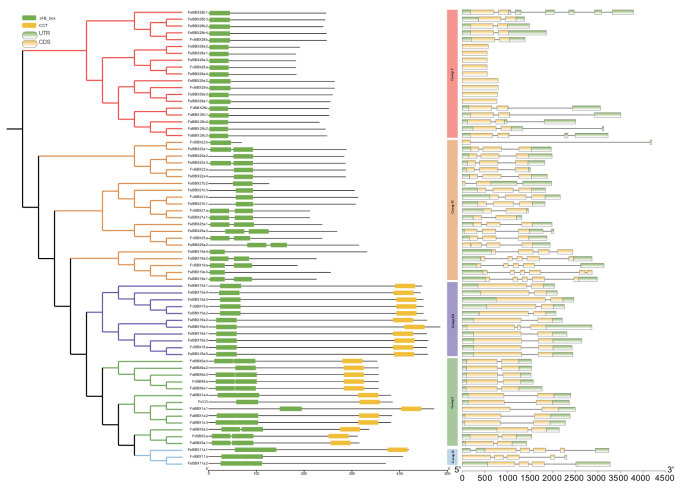
Phylogenetic relationships, gene structures, and conserved domains of the BBX family. Rectangles are the conserve domain in proteins, including B-box domain in green and CCT domain in yellow. Round rectangles are the untranslated region (UTR) region in green and coding sequence (CDS) in yellow.

The conserved domains of the BBX proteins are presented in Figure 2 and Table S2. All identified BBXs contain at least one B-box conserved domain in the N-terminus of proteins. Ten FvBBXs contain only one B-box domain, three of which contain one CCT domain in the C-terminus (FvCO, FvBBX15a, and FvBBX16a). On the other hand, six FvBBXs contain two B-box domains, two of which contain one CCT domain (FvBBX5a and FvBBX6a). Thirty-three FaBBXs contain one B-box domain, including 11 FaBBXs that contain 1 CCT domain. Eighteen FaBBXs contain two B-box domains, nine of which have one CCT domain. In *Arabidopsis*, members of the same phylogenetic group showed similar domain distributions [3]. The members in a same phylogenetic clade showed similar but not exactly identical domain distribution, such as Group I (FvCO-FaBBX1a1), Group III (FvBBX11a-FvBBX11b1), and Group V (FvBBX21b-FaBBX21b1-FaBBX21b2-FaBBX21b3, FvBBX22a-FaBBX22a2). This result indicates that biological functional divergence may exist between members in a same phylogenetic clade. The gene structure diversity of BBX genes in two strawberry species was investigated. The genes in a close phylogenetic relationship share conserved gene structure, especially in Group I and Group III, while the genes in Group IV and Group V present a relative discrepancy of gene structure.

### 2.4. Chromosome Location and Gene Duplication Prediction of BBXs in Strawberry

**Figure 3:**
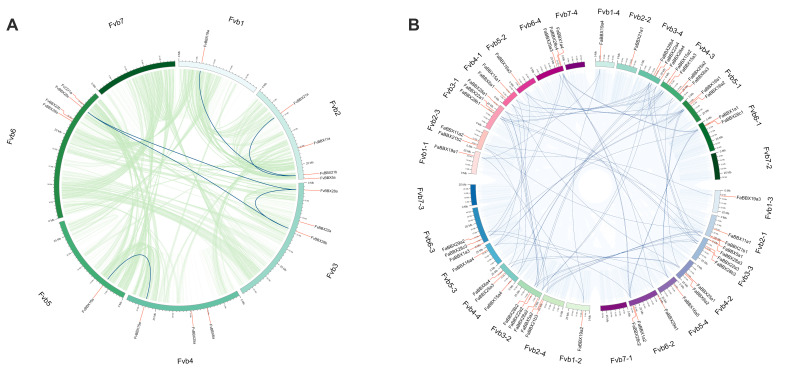
Distributions and duplications of BBXs. (**A**) Distributions and duplications of *FvBBXs* in wild strawberry genome. (**B**) Distributions and duplications of *FaBBXs* in cultivated strawberry genome. The chromosome originated from *F. vesca*-like subgenome (from Fvb1-4 to Fvb7-2), *F. nipponica*-like subgenome (from Fvb1-3 to Fvb7-1), F. iinumae-like subgenome (from Fvb1-2 to Fvb7-3), and F. viridis-like subgenome (from Fvb1-1 to Fvb 7-4).

For both wild strawberry (Figure 3A) and cultivated strawberry (Figure 3B), the BBX genes were unevenly distributed on the chromosomes. In wild strawberry, no BBX gene is located on chromosome 7 (Fvb7). Chromosome 2 (Fvb2) and chromosome 6 (Fvb6) contain four genes, while chromosome 1 (Fvb1) and chromosome 5 (Fvb5) contain only one gene. The BBX genes from cultivated strawberry are similar to those from wild strawberry. The numbers of relative positions on the chromosomes of BBX genes from different subgenomes, including *F. vesca*-like (13 BBXs), *F. nipponica*-like (13 BBXs), F. iinumae-like (13BBXs), and F. viridis-like (12BBXs), are not identical (Figure 3A, Table 1), which supports the distinct origins of subgenomes in cultivated strawberry [16].

**Figure 4:**
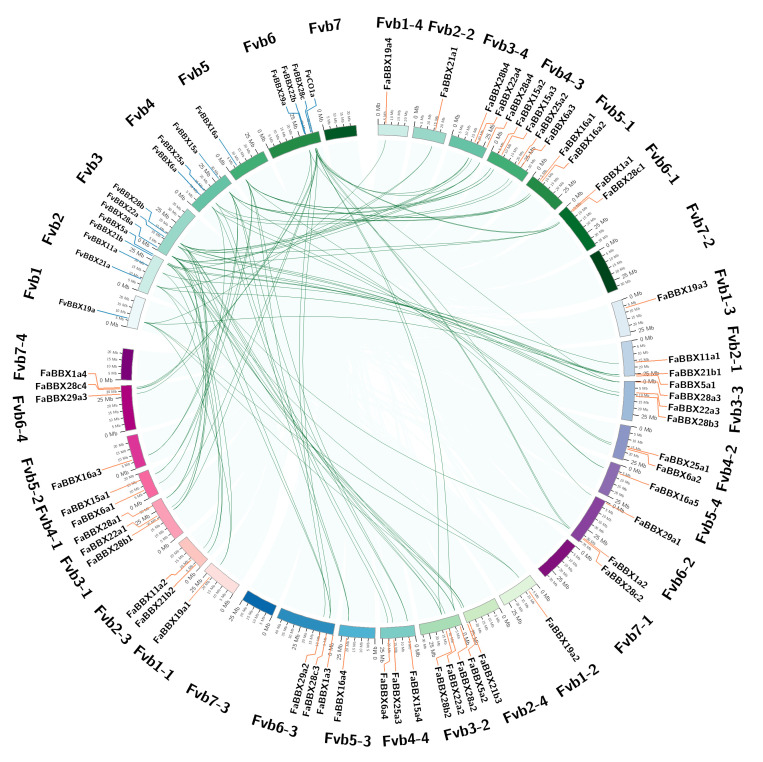
Duplicated gene pairs of BBXs between cultivated strawberry and wild strawberry.

MCScanX classified the duplicated gene pairs into four types, according to the similarity and location of genes [20] (Figure 4, Table S3). Among the *FaBBXs*, 49 genes were labeled as WGD or segmental duplication genes, while two genes were classified as dispersed duplicates. Eight *FvBBXs* were classified as WGD or segmental duplication genes, and the remaining *FvBBXs* were classified into dispersed duplicates. The enrichment analysis shows that WGD or sentimental duplication was the main force driving the expansion of BBX gene family in the two strawberry species. In total, 64 gene duplication pairs were identified within cultivated strawberry, covering all *FaBBXs* except for *FaBBX1a3* and *FaBBX21a1,* which were identified as dispersed duplication genes (Figure 3A, Table S4). Six gene duplication pairs (Figure S3B, Table S5) were identified in wild strawberry, consisting of eight *FvBBX* genes, except for *FvCO, FvBBX5a, FvBBX6a, FvBBX11a, FvBBX22a, FvBBX22b, FvBBX25a,* and *FvBBX29a*. These gene duplication pairs may undergo gene family expansion driven by WGD or segmental duplication.

We performed gene duplicate identification of wild strawberry and cultivated strawberry to understand the evolutionary relationship between *FvBBXs* and *FaBBXs*. As our results show (Figure 4, Table S6), there are 87 gene pairs in the collinearity blocks between the two strawberry species. Two species-specific segmental duplications in cultivated strawberry were observed (Figure 4), namely, *FaBBX15a2/FaBBX15a3* and *FaBBX16a1/FaBBX16a2*. Some of the paired genes were in the same phylogenetic clade, which indicates the reliability and consistency of our phylogenetic analysis and gene duplication analysis.

**Figure 5:**
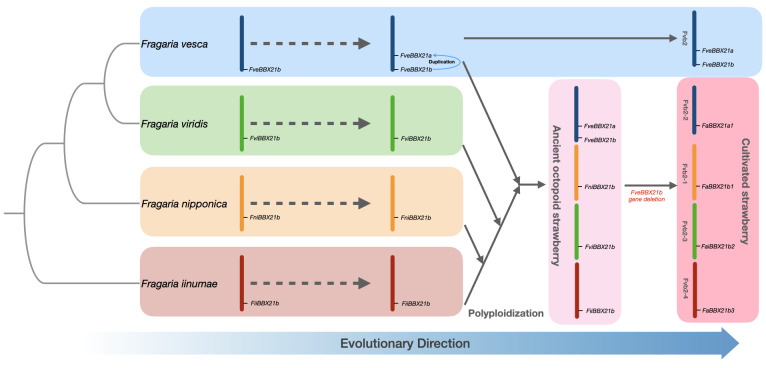
The putative evolutionary route of four paralogs of *FaBBX21*. The bars in four colors represent the second chromosome of four progenitors, namely, *F. vesca* (dark blue), *F. viridis* (green), *F. nipponica* (yellow), and *F. iinumae* (dark red). The blocks of different colors are different species during the evolutionary history of cultivated strawberry.

*FaBBX21a1* from the *F. vesca*-like subgenome was identified as a dispersed duplication gene in the cultivated strawberry genome. Two orthologs (*FvBBX21a* and *FvBBX21b*) were found in wild strawberry in collinearity blocks between cultivated strawberry and wild strawberry (Figure 4). Furthermore, there was a paralogous gene pair of *FvBBX21b* and *FvBBX21a* in collinearity blocks in the wild strawberry genome (Figure 3A). In Table 1, only one ortholog of *FvBBX21a* was from the *F. vesca*-like subgenome, while three orthologs of *FvBBX21b* were located on the other three subgenomes. In addition, the second chromosome (Fvb2-2) of the *F. vesca*-like subgenome in cultivated strawberry was found to be 24.8 Mbp, which is shorter than the corresponding second chromosome (29.4 Mbp) in the wild strawberry genome, implying a deletion or translocation of chromosome 2 (Fvb2-2) of the cultivated strawberry genome during evolution. Taken together, we proposed a putative evolutionary route of *FaBBX21s* (Figure 5). Our inference implies differences in the function of four paralogs of *FaBBX21s* in cultivated strawberry. It is also unknown as to whether there is functional divergence between *FvBBX21a* and *FvBBX21b*.

Gene duplication events may provide a source for functional divergence and evolution. The ratio of the nonsynonymous substitution rate (denoted as *Ka*) to the synonymous substitution rate (denoted as *Ks*) was used as an index to identify the selection pressure of duplicated gene pairs within species. Subfunctionalized daughter copies with transcriptional divergence across cell types or tissues are expected to undergo purifying selection (*Ka/Ks* < 1), whereas nonfunctionalized daughter copies undergo positive selection (*Ka/Ks* > 1) [21]. The *Ka/Ks* values of each gene pair within species were calculated. The intraspecies gene pairs of the two strawberry species are under purifying selection according to the results (Figure S4, Table S7). For wild strawberry, the *Ka/Ks* distribution of the BBX gene family showed no difference with *Ka/Ks* value at genome wide level (*p*-value = 4.412e-1). However, the distribution of *Ka/Ks* of the BBX gene family from the two strawberry species was found to be significantly different (Table S8). This may be caused by the complex origin of cultivated strawberry. In wild strawberry, all gene pairs of collinearity underwent purifying selection after gene duplications (*Ka/Ks* < 1). In cultivated strawberry, *Ka/Ks* values of five duplicated gene pairs were under the adapt selection (*Ka/Ks > 1*), including gene pairs of *FaBBX29a1-FaBBX29a2, FaBBX28c2-FaBBXc3, FaBBX25a1-FaBBX25a2,* and *FaBBX21b1-FaBBX21b3*.

### 2.5. Cis-Reglatory Elements in Promoters of BBX Genes

The promoter region is critical for gene expression regulation. We predicted *cis*-regulatory elements on promoters. Besides the core element of the basal transcriptional machinery in higher plants, such as the TATA box, sixty types of *cis*-regulatory elements were identified in the BBX genes from two strawberry species (Table S9, Figure S5). We further classified them into 20 groups according to their functions annotated by PlantCare and previous studies [22,23,24,25,26,27,28,29,30]. The light response element was the largest category, occupying 29.79 and 27.18% in wild strawberry and cultivated strawberry, respectively (Figure S6, Tables S10 and S11). This result indicates that the BBX genes may play various roles in the light signal transduction pathway. Furthermore, ABA response-related elements are also widely distributed on the promoters of BBX genes, since the percentages of ABA response-related elements are 11.94 and 13.16% in wild strawberry and cultivated strawberry, respectively, which indicates that the BBX genes may participate in the stress response or fruit ripening processes in strawberry. In addition, MYB-related elements are widely distributed in the promoters of BBXs in our study.

**Figure 6:**
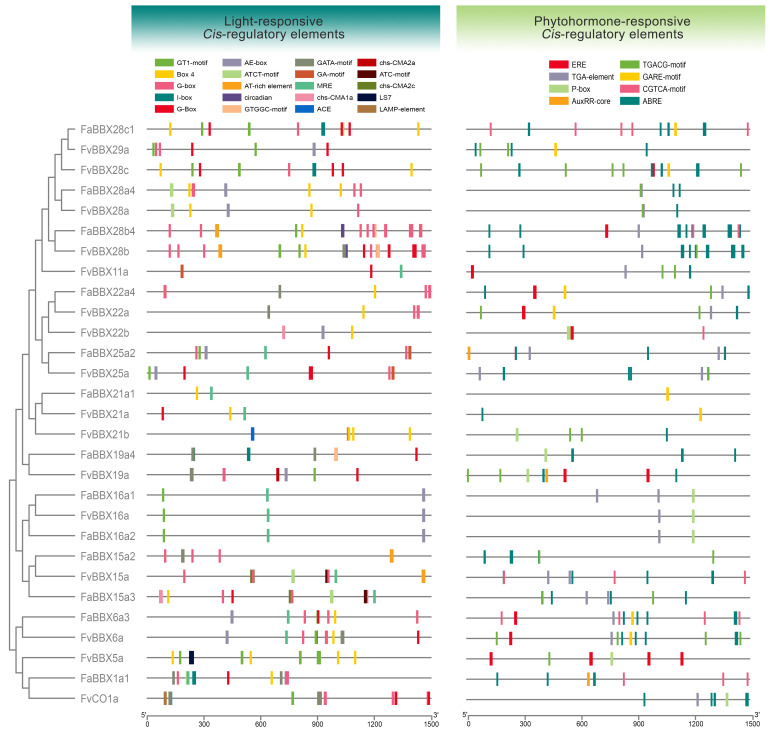
A comparative illustration of *cis*-regulatory elements on the promoter of *FvBBXs* from wild strawberry and *FaBBXs* from the *F. vesca*-like subgenome of cultivated strawberry.

To provide evolutionary information about the promoter region, we compared the arrangement of the light-responsive *cis*-regulatory element and phytohormone-responsive *cis*-regulatory element on the promoters of *FvBBXs* and *FaBBXs* on the *F. vesca*-like subgenome. As shown in Figure 6, a similar distribution of *cis*-regulatory elements was observed among most orthologs, such as *FaBBX28c1-FvBBX28c*, which indicates that they are subjected to similar transcriptional regulatory mechanisms.

The arrangement of *cis*-regulatory elements on the promoter of *FvBB16a* and two orthologs (*FaBBX16a1* and *FaBBX16a2*) shows clear conservatism, which supports our speculation that *FaBBX16a1* and *FaBBX16a2* are derived from segmental duplication events. In contrast, differences in the distribution of *cis*-regulatory elements on *FaBBX15a2* and *FaBBX15a3* suggest that they are perhaps derived from different progenitors. *FaBBX15a2*, which is more different from *FvBBX15a*, may undergo an exchange of the fragment of subgenomes, which results in the gene translocation from other subgenomes to the *F. vesca*-like subgenome. *FvCO1* was previously identified as a key regulator of flowering time in wild strawberry [10]. In *Arabidopsis*, *AtCO* is regulated by various transcription factors such as AtCDF1 [31]. A dramatically discrepant *cis*-regulatory element distribution was found between *FvCO1* and *FaBBX1a1,* which implies a divergent regulatory mechanism between wild strawberry and cultivated strawberry. This divergence may be due to the domestication of cultivated strawberry for longer flowering time and higher fruit yield.

### 2.6. The Expression of BBXs in Strawberry

**Figure 7:**
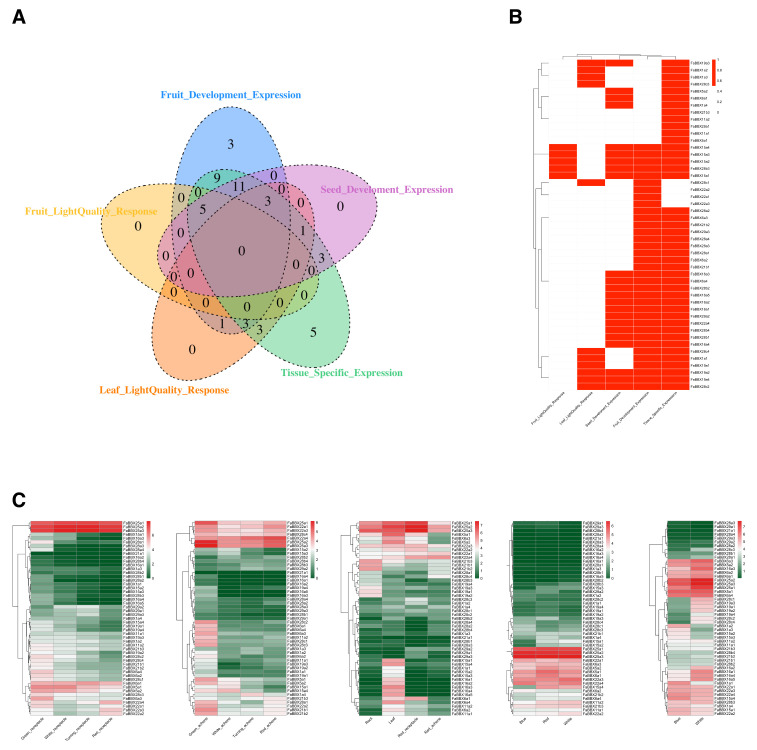
Gene expression of *FaBBXs*. (**A**) Venn diagram of differentially expressed *FaBBXs*. (**B**) A heat map diagram of differentially expressed *FaBBXs*. (**C**) Heat map of the gene expression level of FaBBX genes according to RNA-seq. TPM was used for normalization.

We further analyzed the expression pattern of *FaBBXs* in the development stages of receptacle and achene, the different tissues, fruits under different quality treatments, and leaves under different light quality treatments (Figure 7C and Figure S7).

We identified 47 differentially expressed *FaBBXs* (Figure 7A,B), which indicates that the BBXs in cultivated strawberry participated in various transcriptional regulation networks, including the response to external environment and progression of development. Among differentially expressed *FaBBXs*, several *FaBBXs,* including *FaBBX19a3*, *FaBBX15a1*, *FaBBX15a2*, *FaBBX15a3*, *FaBBX15a4*, *FaBBX28b3*, *FaBBX19a2*, *FaBBX19a4* and *FaBBX28c2,* participate in multiple regulatory pathways, such as light signaling and fruit development. Generally, most of the genes in the same phylogenetic clade show similar expression patterns (Figure 7B and Figure S7). However, there are conflicting expression patterns of *FaBBXs* of the same phylogenetic clade. For example, *FaBBX28c3* and *FaBBX28c4* have a close phylogenetic relationship with each other, whereas their expression shows diverge in the comparison between roots and leaves. This divergent expression pattern reflects divergence of the gene functions.

In our results, the expression levels of 16 *FaBBXs* are significantly different under light quality treatments (Figure 7A and Figure S7). Five *FaBBXs* were expressed differently in the fruit under different light quality treatments, while 11 *FaBBXs* responded to light quality treatment in the leaves under blue light treatment. These genes are homologs of *FaBBX1a*, *FaBBX15a*, *FaBBX19a*, and *FaBBX28c*.

### 2.7. qRT-PCR Analysis of FaBBXs

**Figure 8:**
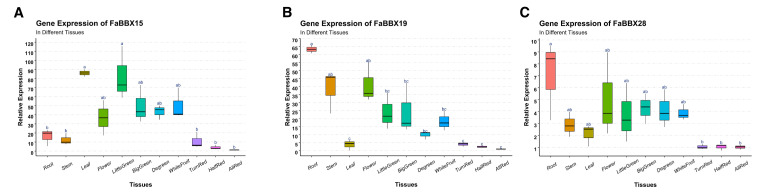
Box plots of the gene expression of three FaBBX genes. (**A**) Expression pattern of *FaBBX15a* in different tissues and different developmental stages of strawberry fruit. (**B**) Expression pattern of *FaBBX19a* in different tissues and different developmental stages of strawberry fruit. (**C**) Expression pattern of *FaBBX28c* in different tissues and different developmental stages of strawberry fruit. The significance are annotated by letters.

RNA-seq analysis provides a global view of the expression of *FaBBXs*. On the basis of the RNA-seq analysis, we selected three light-responsive *FaBBXs*, namely, *FaBBX15a*, *FaBBX19a*, and *FaBBX28c*, for further expression analysis using quantitative real-time PCR (qRT-PCR) with a focus on the expression level of different tissues and the stages of fruit development (Figure 8).

As expected, all selected *FaBBXs* were expressed in different tissues and showed tissue-specific expression patterns. An expression peak of *FaBBX15* was observed in the leaf tissue of strawberry. A continuously decreasing expression pattern of *FaBBX15* was shown with the ripening process of strawberry fruit. There was a highly similar expression pattern between *FaBBX19* and *FaBBX28*. Both *FaBBX19* and *FaBBX28* showed the highest expression in root tissue. Moreover, a similar decline in the expression levels of *FaBBX19* and *FaBBX28* during different developmental stages of strawberry fruit was observed in our results, which suggests the potential similarity of gene functions between *FaBBX19* and *FaBBX28*.

### 2.8. Subcellular Localization and Transactivation of FaBBXs

**Figure 9:**
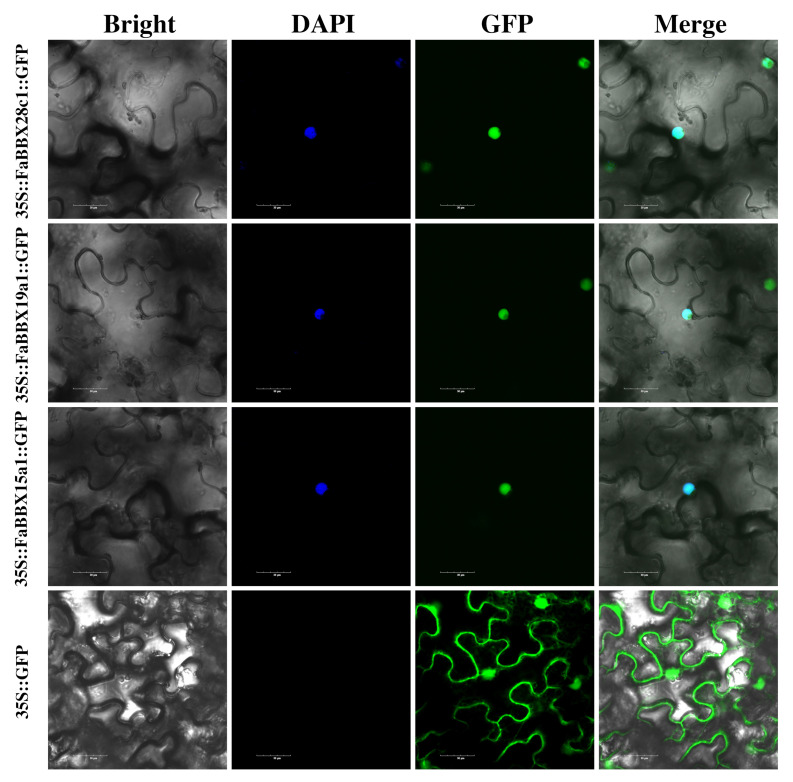
Subcellular localization of FaBBX proteins. The FaBBXs::GFP fusion protein was observed in tobacco epidermal cells. Bar = 30 μm.

To identify the transcription factor features of the three selected FaBBXs, we performed subcellular localization analysis and transactivation analysis. We cloned coding sequences of three FaBBX proteins (Table S12) for plasmid constructs encoding a fusion protein containing BBX protein and green fluorescent protein (FaBBX::GFP) driven by the 35S promoter (sequence of plasmid is listed in File S). Each subcellular localization vector was transiently expressed in tobacco leaves. The empty vector of GFP, which was used as a positive control, resulted in a diffuse distribution of the green fluorescence signal of GFP in the entire cells. The GFP fluorescence signals of FaBBX::GFP fusion proteins were predominantly localized in the nucleus (Figure 9). The subcellular localization results indicate that the selected FaBBXs are nucleus-localized proteins.

**Figure 10:**
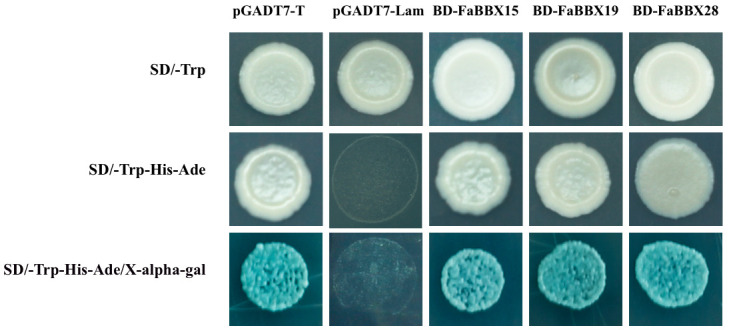
Transactivity assay of FaBBX proteins in yeast cells.

The transcriptional activities of FaBBXs in yeast cells were analyzed. The tested transformed yeast lines harboring BD-FaBBX vectors grew well on SD/-Trp media as the positive control yeast (Figure 10). All yeast cell lines grew on triple dropout medium plates. The yeast lines were dropped on triple dropout medium that contained X-α-Gal to detect the activation of the MEL1 gene in yeast cells. The yeast lines showed a blue stain on the plate. Taken together, FaBBX15, FaBBX19, and FaBBX28 showed transactivation ability to promote the reporter system in yeast.

### 2.9. Overexpression FaBBX28c1 in Arabidopsis

**Figure 11:**
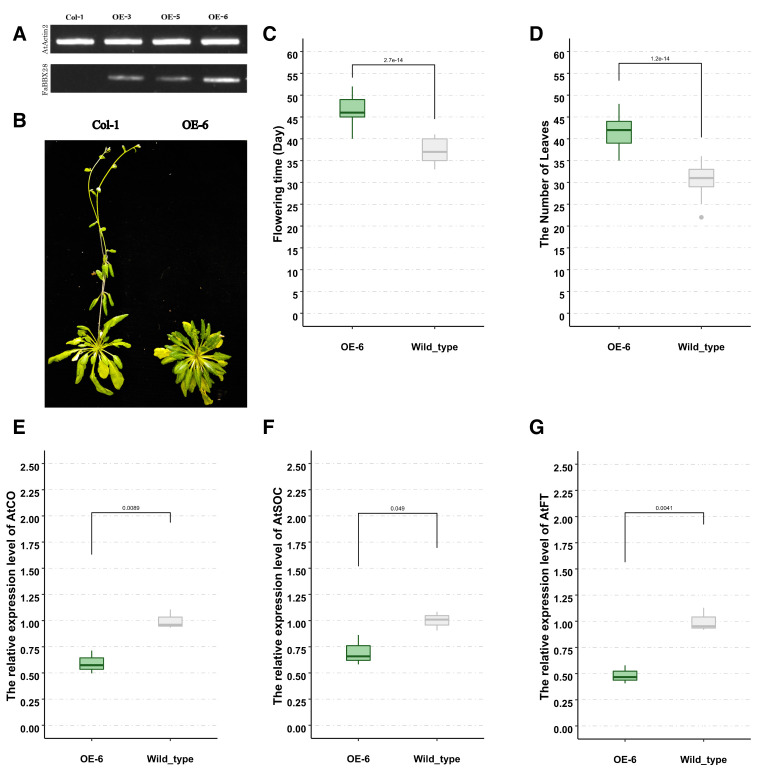
Genotype and phenotype of transgenic *Arabidopsis*. (**A**) Identification of the gene expression levels of *FaBBX28c1* in transgenic *Arabidopsis* lines using semi-qRT-PCR. (**B**) Phenotype comparison of wild-type and transgenic *Arabidopsis* overexpressing *FaBBX28c1*. (**C**) Box plot of flowering time of wild-type and overexpression *Arabidopsis* lines under long-day photoperiodic condition. (**D**) Box plot of the number of rosette leaves of wild-type and overexpression *Arabidopsis* lines under long-day photoperiodic condition. (**E**) Box plot of the expression level of *AtSOC1* in wild-type and overexpressing *Arabidopsis* lines. (**F**) Box plot of the expression level of *AtFT1* in wild-type and overexpressing *Arabidopsis* lines. (**G**) Box plot of the expression level of *AtCO* in wild-type and overexpressing *Arabidopsis* lines.

Our research shows that *FaBBX28c1* may play a role in the flowering time regulation of strawberry seedlings under blue light treatments (Figure S7). To further investigate the function of *FaBBX28c1*, we constructed *FaBBX28c1* overexpression lines using *Arabidopsis* Col-0 as a background. The validation of transgenic *Arabidopsis* using semi-qRT-PCR showed that three independent lines were obtained with the highest expression level in OE-6 line (Figure 11A). Therefore, we selected OE-6 line for further phenotypic analysis. The transgenic *Arabidopsis* plants showed a phenotype of late flowering time under long-day photoperiodic condition (Figure 11B,C and Figure S8). In addition, there were more transgenic *Arabidopsis* rosette leaves than the wild type (Figure 11D and Figure S9). These results show that FaBBX28 may regulate the flowering time and vegetative growth of *Arabidopsis*.

To confirm the phenotype of flowering time, we performed qRT-PCR to examine three crucial genes in the photoperiodic flowering time pathway. The expression levels of *AtCO*, *AtFT*, and *AtSOC1* were downregulated in the overexpression lines (Figure 11E–G). These results suggest that FaBBX28c1 may function as an upstream regulator of the CO-FT-SOC1 pathway in *Arabidopsis*.

### 2.10. Activity of proFaBBX28c1

**Figure 12:**
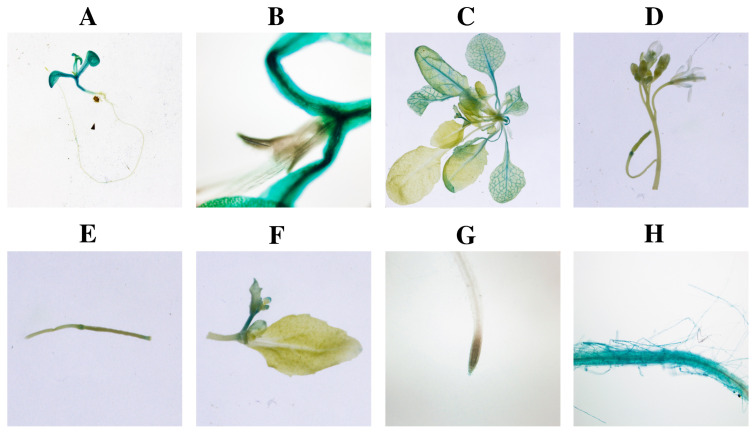
GUS staining in transgenic *Arabidopsis* harboring *proFaBBX28c1*::GUS report system. An overview of GUS staining of transgenic *Arabidopsis* plants (**A**). The GUS staining in mature leaves of transgenic *Arabidopsis* plants (**B**,**C**). The GUS staining in the flower of transgenic *Arabidopsis* (**D**). The GUS staining in the tip and base of the siliques of the transgenic *Arabidopsis* plants (**E**). The GUS staining in the buds of transgenic *Arabidopsis* plants (**F**). The GUS staining in root of the transgenic *Arabidopsis* (**G**,**H**).

The qRT-PCR analysis showed a tissue-specific expression pattern of the *FaBBX28c* gene. To obtain more details on the gene expression of *FaBBX28c1*, we cloned a 1743 bp upstream sequence of *FaBBX28c1* (*proFaBBX28c1*) from the start codon of *FaBBX28c1*. ProFaBBX28c1 was further inserted into pCambia1301 to drive the GUS gene as a reporter system. The plasmid vector containing the reporter system was further transformed into wild-type *Arabidopsis*. As Figure 12 shows, GUS staining of transformed *Arabidopsis* plants was observed in different tissues. The *proFaBBX28c1*::GUS expression was observed in the mature cotyledon of *Arabidopsis* seedlings. However, no GUS staining was detected in the young leaves of *Arabidopsis* in either young seedlings or mature plantlets. In addition, the GUS detected in leaves was clearly observed and distributed in the vascular of leaves. In maturing siliques, *proFaBBX28c1*::GUS expression was detected in the portions of the tip and base of the siliques. Significant GUS staining was detected in the flower bud tissue. However, in mature flowers, GUS staining was hardly observed. Our previous gene transcription level analysis shows that the highest expression of *FaBBX28* was detected in root tissue. To our surprise, there was no obvious GUS staining in the root tissue compared with other tissues. Therefore, we presumed that the expression of *FaBBX28* was inducible by the soil environment, such as drought stress. The GUS reporter system was not induced in the roots when the seedlings grew in MS medium. However, for some parts of the root with visible GUS staining (Figure 12H), these parts of the root may have been exposed to air and under an inducible stress environment.

## 3. Discussion

The BBX gene family are widely distributed in plants as a class of transcription factors involved in various physiological processes, such as flowering time regulation, light signal transduction, and stress signaling pathways [2]. During the past 10 years, BBX gene families in various species have been identified with a systematic bioinformatics method. Previous studies have shown that the number of BBXs varies among different species [16,32]. In the present study, 16 *FvBBXs* and 51 *FaBBXs* were identified and classified into five groups, which is consistent with previous studies [3,33,34].

Increasingly, studies on high plant genome sequencing have shown that the evolution of gene families is associated with gene duplication. Repeated episodes of small-scale (such as tandem gene duplication) and large-scale (such as whole-genome duplication (WGD) and segmental duplication) gene duplication events are two major types of gene duplication events during the evolution of the plant genome [35]. *Angiosperm* (flowering plant) genomes have undergone recurring whole genome duplications over the past ≈200 million years [36]. In *Arabidopsis*, three whole-genome duplications were directly responsible for >90% of the increase in transcription factors, signal transducers, and developmental genes in the last 350 million years [37]. The gene duplication event may cause the expansion of 67 *MdBBX* genes in the apple genome, which results in more BBX in apple than in other *Rosacea* plants. Compared to the *MdBBXs*, the BBX gene family from two strawberry species is less abundant than apple, which is consistent with the previous report [34]. This may be a result of the recent WGD event, which is specific for the apple genome and pear genome [38]. In the present study, the enrichment analysis of duplication events shows that the large-scale duplication event (WGD and segmental duplication) is the main force that drives the expansion of the BBX gene family in wild strawberry. Cultivated strawberry was reported to be the allo-octploid descendant of the merger of four diploid progenitor species into a single nucleus [15]. In our results, almost gene pairs of *FaBBXs* were identified to be driven by WGD and segmental duplication. The polypoid hybridization event during the evolution of cultivated strawberry could be the reason for this phenomenon because the MCScan algorithm inferred duplicated gene pairs on the basis of the similarity and location of genes, which could overestimate the rate of genes originating from large-scale duplication events [20].

Gene duplication was observed in wild strawberry, such as *FvBBX21a/FvBBX21b*, which suggests a family expansion of *FvBBXs* in wild strawberry driven by gene duplication. Gene loss events involving paralogs of *FaBBX21s* in cultivated strawberry were found and can be evolutionarily significant in polyploid plants [39,40,41]. In some phylogenetic clades, such as *FvBBX11a-FaBBX11a2*, prologues cannot be found from all subgenomes. This is similar to a previous report about the *FaMLO* gene family in cultivated strawberry, which attributed this phenomenon to the genome variation of the progenitors [40]. However, gene loss during the evolution of octoploid strawberry can also be the reason. Therefore, more genome information about the other three diploid strawberries is needed for further explanation. Unique segmental duplication gene pairs, such as *FaBBX16a1* and *FaBBX16a2*, were found in *F. vesca*-like subgenome in cultivated strawberry. Since the *F. vesca*-like subgenome is the single dominant subgenome [15], gene loss and gain may affect the unique traits of cultivated strawberry. A putative gene translocation (*FaBBX15a2* and *FaBBX15a3*) from other subgenomes to the *F. vesca*-like subgenome was found, which provides evidence of the dominance of the *F. vesca*-like subgenome during homologous chromosomes exchange [15,42]. A recent study showed that PbBBX18, which is a homolog of the BBX21 protein, participated in anthocyanin biosynthesis in the peel of pear fruit [43]. On the basis of our result, we propose a divergent evolution process of BBX21, which can affect the fruit quality of the two strawberry species. Therefore, further comparative analyses about two homologs of FvBBX21s and FaBBX21a1 are required. However, the biological significance of these family expansion events for the flowering regulation mechanism of strawberry need to be further explored, since functional studies of the above genes in plant flowering regulation remain scarce.

BBX genes are reported to play diverse functional roles in plant biological processes [4]. Increasing evidence has demonstrated that BBXs show special gene expression patterns related to their function. *PpBBX16* from pear (*Pyrus pyrifolia*), which was identified as a positive regulator of anthocyanin accumulation, showed an expression peak after light treatments [44]. MdBBX37, whose gene expression was repressed by light, interacted with two key positive regulators of anthocyanin biosynthesis and negatively regulated anthocyanin biosynthesis [45]. In addition, gene expression is regulated by *cis*-regulatory elements. *Cis*-regulatory elements play roles as molecular switches contributing to transcript regulation and participates in complex gene networks [46]. The light response element is enriched on the promoter region of BBX genes from strawberry. Phytohormone-responsive cis-regulatory elements are also a class of cis-regulatory elements widely distributed in the promoter regions of BBX genes. In addition, previous research demonstrates that BBX genes participate in light signaling [4]. Most *FaBBXs* show differential expression under different light qualities and during the development of strawberry, which corresponds to the findings on the other plants. The qRT-PCR analyses of three selected *FaBBXs* show tissue-specific expression patterns. For *FaBBX15*, the expression peaks were observed in the leaf and little green stage of strawberry fruit. *PhCOL16* from petunia (*Petunia hybrida*) is associated with chlorophyll content and involved in chlorophyll accumulation [47]. Therefore, we deduce that homologs of BBX15 in strawberry may play roles in the regulation of chlorophyll biosynthesis in leaves and degreen processes during the development of strawberry fruits. A similar expression pattern of *FaBBX19a* and *FaBBX28c* was observed. It is well understood that *AtBBX19* plays dual roles in the regulation of flowering time in *Arabidopsis* and tolerance to drought stress in chrysanthemum [9,48]. However, the function of *AtBBX28* remains contentious [6,7]. In the present study, the highest expression of *FaBBX19* and *FaBBX28* was also observed in the root tissue. This may imply a similarity of gene functions between homologs of *FaBBX19a* and *FaBBX28c* in the regulation of tolerance to drought stress. Focusing on the function of homologs of *FaBBX28c* in strawberry in more detail is necessary.

The gene promoters ligated to the GUS reporter can be used in a further investigation of spatial and temporal expression patterns [49]. Here, we provided a better understanding of the spatial expression of *FaBBX28c1* in transgenic *Arabidopsis* plants using the *proFaBBX28c1*::GUS reporter system. The qRT-PCR results show that the expression of FaBBX28 in leaves was much lower than that in other tissues. However, GUS staining was observed in the old leaves but not in the young leaves. The young leaf sample for qRT-PCR is the reason for the difference between the two results. Additionally, a previous report on the function of *FvFT1*, which is a regulator of flowering time of wild strawberry, shows that *FvFT1* has the highest expression level in old leaves, and no expression or weak expression was observed in young leaves [50]. The similar spatial expression between *FvFT1* and *FaBBX28* leads to a reasonable assumption of a functional relationship of the two genes.

**Figure 13:**
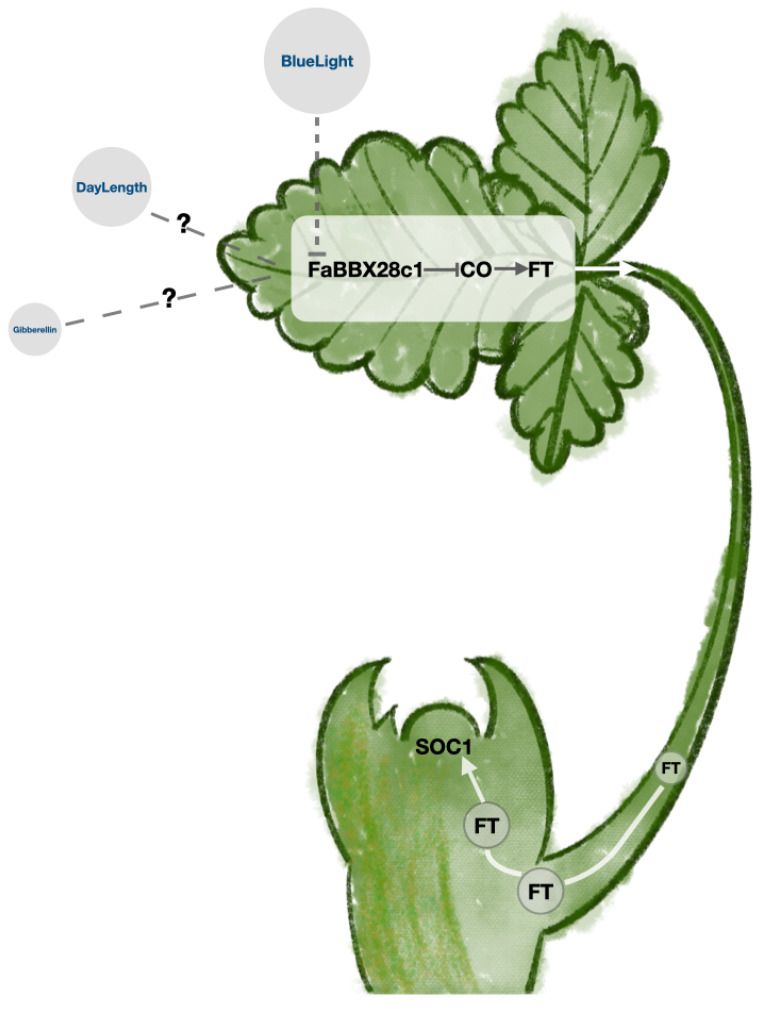
Function of FaBBX28c1 in the regulation model of flowering time. *FaBBX28c1* may function as an upstream negative regulator of the CO gene. The expression level of *FaBBX28c1* was repressed by blue light treatments.

In strawberry, vegetative and generative developmental programs are tightly connected by flowering time, which is a key of the transition from vegetative to reproductive growth during the plant life cycle [51,52]. An understanding of the genetic mechanisms underlying flowering time in strawberry could facilitate the strawberry breeding work. The TERMINAL FLOWER1 (*FvTFL1*) was demonstrated as the basis of the flowering behavior contrast between the seasonal flowering wild strawberry with the perpetual flowering accessions [17]. In cultivated strawberry, *FaTFL1* was further used as a breeding target for specific flowering characteristics [50]. In the present study, the transgenic plants overexpressing *FaBBX28c1* showed a phenotype of late flowering under long-day photoperiodic condition. In addition, the number of rosette leaves in transgenic plants significantly increased. The balance of vegetative growth and reproductive growth of plants is regulated by genetic background and environmental conditions such as day length and temperature. Our results suggest that *FaBBX28c1* may play roles in the balance of vegetative growth and reproductive growth in *Arabidopsis* by regulating flowering time. A flowering regulation pathway consisting of *AtCO-AtFT-AtSOC* has been established in *Arabidopsis* [53,54]. Our results show a downregulation of those genes in the overexpression lines, which suggests that *FaBBX28c1* may function as an upstream negative regulator of the pathway (Figure 13), which is similar to its homologs in *Arabidopsis* [6].

In addition, recent works from strawberry also confirm the pathway of FvCO-FvFT1-FvSOC1 in the control of flowering time [10,55]. Blue light affects the regulation of flowering time in both wild strawberry and cultivated strawberry [18,19]. Transcriptome analysis of cultivated strawberry under blue light quality treatments enriched the DEGs into BBX gene family [56]. The result of this study showed that *FaBBX28c1* was downregulated under blue light treatment (Figure S7). The blue light treatment may repress the expression level of *FaBBX28c1* to further promote the flowering of cultivated strawberry. The function of *FaBBX28c1* in the flowering time changes due to the blue light treatment, including its role in the blue light signaling and the functional relationship with the known flowering time regulation pathway, should be further explored.

## 4. Materials and Methods

### 4.1. Identification of BBX Family Members in the Strawberry Genome

The genome data of wild strawberry (*Fragaria vesca* ssp ‘Hawai 4’) v4.0.a1 [57] and cultivated strawberry (*Fragaria ananassa cv* ‘Camarosa’) v1.0.a1 [15] were retrieved from the GDR database [58]. An HMM profile (hidden Markov model) of the B-box conserved domain (PF00643) was download from the Pfam database [59]. A search against the genome protein database was conducted with the default parameters setting using HMMER software (v3.2) [60]. The output putative sequences of proteins were further confirmed by the Pfam online tool (http://pfam.xfam.org/, accessed on 1 July 2021). The redundant sequences were removed to retain the longest protein sequence among different transcript isoforms from a same gene.

### 4.2. Phylogenetic Analysis and Nomenclature of BBXs

The sequences of BBX proteins from wild strawberry, cultivated strawberry, and *Arabidopsis* were used for the construction of the phylogenetic tree. The sequences were aligned by the Mafft software (Version 5) [61]. An unroot tree was subsequently generated by IQ-tree (Version 2.0) with JTT+I+G4 substitution model [62]. Both bootstrap test and approximate likelihood ratio test were set as 1000 times. The BBX genes were named following the nomenclature scheme proposed by Khanna [3]. For the genes in a same clade, the names followed the order of the genome annotation to label with lower case letters and numbers at the end of gene names. The phylogenetic trees were visualized with iTOL version6 [63].

### 4.3. Protein and Gene Structure Characteristics

Physical and chemical parameters of BBX proteins were predicted by a script of Bioperl toolkit (File S) [64]. The Pfam web tool was employed to identify the conserved domains in proteins with default parameter settings. The gene structures of *FvBBXs* and *FaBBXs* were visualized by TBtools [65].

### 4.4. Chromosome Location and Gene Duplication Prediction of BBXs in Strawberry

The physical gene locations of BBXs in the strawberry genome were extracted from the genome annotation. The MCScanX software was used to identify the duplication of BBX genes interspecies (between different species) or intraspecies (within the same species). An enrichment analysis was used to identify the association between the number of gene family and a particular genome-wide duplication mode with Fisher’s exactly test [20]. The *Ka* and *Ks* values of duplicated gene pairs of WGD/segmental or tandem duplicates were calculated using a described pipeline [41]. The collinear and tandem relationships and gene locations of BBX genes were visualized by Circos software (version 0.69-9) [66].

### 4.5. Cis-Regulatory Element Prediction and RNA-seq Analysis

The promoter sequences of BBXs, which are 1500bp upstream of transcription start sit, were retrieve by Seqkit software (v0.16.0) [67]. The promoter sequences were submitted to PlantCare for identification of *cis*-regulatory elements located on the promoter sequences [68]. The expression level was analyzed using RNA-Seq data, which was reported by previous research with the accession of PRJEB12420, PRJNA734001, and PRJNA698363 [56,69,70]. The transcriptome data were processed with the Hisat2-StringTie pipeline [71]. Expression levels were calculated and normalized as transcript per million (TPM). DEseq2 was employed as a statistics tool to identify the DEGs with a criterion of fold change and *p*-value (|log2 fold change|≥2 and *p*-value < 0.05) [72]. The results of gene expression level and DEGs analysis were visualized using tidyverse package (version 1.3.0) under R platform (version 4.0.3) [73].

### 4.6. Plant Materials Cultivation and Treatments

Seedlings of cultivated strawberry (*Fragaria ananassa* cv. ‘Benihoppe’) were grown in a greenhouse of Sichuan Agricultural University, Wenjiang, Sichuan, China. The seedlings were planted in 10 × 10 × 12 cm plastic pots with a 1:4 (volume ratio) mixture of vermiculite and peat soil. Subsequently, the seedlings were transferred to the greenhouse with the condition of 20–28 °C temperature and 50~75% relative humidity in September 2018.

For the gene expression analysis of different tissues of strawberry, we harvested samples from cultivated strawberry, including root, stem, stolon, flower, and fruit. The criteria of seven different development stages of strawberry fruit are defined according to a previous report [74].

Experiments involving *Arabidopsis* were based on the Col-0 ecotype. Seeds of transformed *Arabidopsis* lines were germinated by an exposure at the temperature of 4 °C for 72 h for vernalization. The *Arabidopsis* seeds were sowed into a 1:4 (volume ratio) mixture of vermiculite and peat soil and were watered regularly. The seedlings of *Arabidopsis* were subjected to a normal growth condition with temperature of 22/18 °C (day/night) and 16/8 h light/dark cycle under an artificial light level of 125 μmol/m^2^/s and relative humidity of 40%. The leave for future analysis was sampled when the flower buds appeared. The flowering time and the number of leaves of *Arabidopsis* lines were recorded.

All samples harvested were immediately frozen in liquid nitrogen and stored at −80 °C for downstream analysis.

### 4.7. RNA Extraction and Gene Expression Analysis

The total RNA of all samples was extracted with a modified CTAB method [75]. The quality of RNA was evaluated by electrophoresis on a 1% agarose gel and scanned using a NanoDrop spectrophotometer. One microgram of total RNA was used for a reverse-transcription PCR reaction with Prime-Script^TM^ RT Reagent Kit with gDNA Eraser (Takara, Japan), following the manufacturer’s protocol. The cDNA was used in sequential 20 μL qRT-PCR reaction system as the template on the basis of a SYBR Premix ExTaq^TM^ Kit (Takara, Dalian, China). The qRT-PCRs were performed on the CFX96 real-time PCR system (Bio-Rad, Hercules, CA, USA). Each reaction was performed with three technical replicates. The *FaActin2* and *AtActin2* were used as housekeeping genes for the calculation of relative expression value using the Livak’s method [76]. The primer sequences are listed in File S.

### 4.8. Ectopic Expression of FaBBX28c1 in Arabidopsis

A pair of primers (File S) was designed to clone the coding sequence (CDS) of FaBBX28c1 into the multiple clone site of a modified pCambia1301 plasmid (pCam-bia1301-35SN, File S) using a CloneExpress II One Step Cloning Kit (Vazyme, Nanjing, China). The recombined expression plasmid was transformed into *Agrobacterium tumefaciens* strain GV3101. The *Arabidopsis* plants were transformed by the floral dip method [77]. T1 and T2 progeny were screened on 1/2 MS plates containing 50 mg/L hygromycin-B. The T3 generation was used for the phenotype observation and expression profiling under long-day photoperiodic condition.

### 4.9. proFaBBX28c1 Activity Analysis in Arabidopsis

The promoter sequence of FaBBX28c1 (proFaBBX28c1) was amplified and inserted into the restriction enzyme site in front of β-glucuronidase (GUS) report gene (gus) of pCambia1301 by using CloneEx-press II One Step Cloning Kit (Vazyme, Nanjing, China) (File S) to fuse the promoter of *proFaBBX28c1* and GUS. The plasmid construction of *proFaBBX28c1*::GUS was transformed into *Agrobacterium tumefaciens* strain GV3101 and subsequently transformed into *Arabidopsis* for promoter activity analysis.

T2 progeny seedlings containing *proFaBBX28c1*::GUS reporter were used for GUS staining. The GUS staining was performed following the manufacturer’s instructions described by the β-Galactosidase Reporter Gene Staining Kit (Solarbio, Beijing, China).

### 4.10. Sub-Cellular Localization of FaBBX Proteins

The CDS of FaBBXs were amplified (Table S1) and inserted into a plasmid vector (pYTSL-16), which was modified from pMDC83-35S and pSITE-2NB, resulting in a plasmid vector expressing a fusion protein of FaBBXs::GFP (File S).

The plasmid was further transformed into *Agrobacterium tumefaciens* strain GV3101. The empty vector was used as a control. The plasmids were transiently expressed in the epidermal cells of tobacco (*Nicotiana benthamiana*) leaves as previously described [78]. The 4′,6-diamidino-2-phenylindole (DAPI) staining was used as a nucleus marker. All the fluorescence signals of the samples were detected by a confocal laser scanning microscopy system (FV3000 Olympus, Tokyo, Japan).

### 4.11. Transactivation Activity Analysis of FaBBXs Protein in Yeast

To verify the transcriptional activity of FaBBXs protein in yeast, we constructed pGBKT7-FaBBX vectors expressing the fusion proteins of BD-FaBBXs using a similar method as aforementioned (Table S1). The positive vector was confirmed and transferred into yeast strain Y2HGold component cell. The positive (pGADT7-T) and negative (pGADT7-lam) plasmids were set up as controls. The transformed yeast cells for the auto-activation test were cultured in liquid synthetic drop-out (SD)/-Trp 3 days at 30 °C. The transformants were stripped onto SD/-Trp-His-Ade and SD/-Trp-His-Ade/X-alpha-gal plates.

## 5. Conclusions

In the present study, we identified 53 *FaBBXs* and 16 *FvBBXs* in two strawberries. Evolutionary analysis shows that large-scale duplication events are the main force driving the expansion of the BBX gene family in strawberry. Gene translocation, gene duplication, and loss events were found in the *F. vesca*-like subgenome of cultivated strawberry and can affect the important traits of cultivated strawberry. The BBX genes in cultivated strawberry participate in light signaling, which can participate in the regulation of flowering time. An *Arabidopsis* line overexpressing *FaBBX28c1* demonstrates that *FaBBX28c1* may function as an upstream regulator of the CO protein. Taken together, our results provide useful evolutionary information and expression profiles of the BBX gene family in strawberry. The primary functional identification of *FaBBX28c1* highlights the role of *FaBBXs* in regulating the flowering time.

## Figures and Tables

**Table 1:** Number of BBX genes from two strawberry species in phylogenic clades.

Phylogenic Clade ^1^	Number of *FvBBXs*	Number of *FaBBXs*	Number of *FaBBXs* from Subgenomes			
			*F. vesca*-like	*F. niponica*-like	*F. Iimae*-like	*F. viridis*-like
*FvCO-FaBBX1a4*	1	4	1	1	1	1
*FvBBX5a-FaBBX5a2*	1	2	0	1	1	0
*FvBBX6a-FaBBX6a4*	1	4	1	1	1	1
*FvBBX11a-FaBBX11a2*	1	2	0	1	0	1
*FvBBX15a-FaBBX15a4*	1	4	2	0	1	1
*FvBBX16a-FaBBX16a5*	1	5	2	1	1	1
*FvBBX19a-FaBBX19a4*	1	4	1	1	1	1
*FvBBX21a-FaBBX21a1*	1	1	1	0	0	0
*FvBBX21b-FaBBX21b3*	1	3	0	1	1	1
*FvBBX22a-FaBBX22a4*	1	4	1	1	1	1
*FvBBX22b*	1	0	0	0	0	0
*FvBBX25a-FaBBX25a3*	1	3	1	1	1	0
*FvBBX28a-FaBBX28a4*	1	4	1	1	1	1
*FvBBX28b-FaBBX28b4*	1	4	1	1	1	1
*FvBBX28c-FaBBX28c4*	1	4	1	1	1	1
*FvBBX29a-FaBBX29a3*	1	3	0	1	1	1

^1^ The phylogenic clade is according to Figure 1 from wild strawberry gene tips to cultivated strawberry gene node.

## Data Availability

The data presented in this study are available on request from the corresponding author.

## Supplementary Materials

The following are available online at https://www.mdpi.com/article/10.3390/ijms222111766/s1.
