# Supplementary material for: An Evolutionary Analysis of B-Box Transcription Factors in Strawberry Reveals the Role of FaBBx28c1 in the Regulation of Flowering Time
Source: Int J Mol Sci. 2021 Oct 29;22(21):11766. doi: 10.3390/ijms222111766 (PMC8583817; doi:10.3390/ijms222111766)
Supplement: Supplementary file 1 [file ijms-22-11766-s001.zip › SFiles/Figure S1.pdf]

An alignment of mRNA sequence of *FvCO*(gene04172) and *FvBBX1*(FvH4_6g45860)

Reference sequence (1): FvH4_6g45860
Identities normalised by aligned length.
Colored by: identity

```
                                 cov    pid   1 [         .         .         .         .         :         .         .         . 80
1 FvH4_6g45860              100.0% 100.0%      CTGGATTCTCGTTCCACTTTGCCGCTCTGCCTACAGTTACACCCCCTCTCCCTCCTCCTTCCCTCCATAACTCCATTATT
2 gene04172-v1.0-hybrid     100.0% 100.0%      CTGGATTCTCGTTCCACTTTGCCGCTCTGCCTACAGTTACACCCCCTCTCCCTCCTCCTTCCCTCCATAACTCCATTATT
  consensus/100%                               CTGGATTCTCGTTCCACTTTGCCGCTCTGCCTACAGTTACACCCCCTCTCCCTCCTCCTTCCCTCCATAACTCCATTATT
  consensus/90%                                CTGGATTCTCGTTCCACTTTGCCGCTCTGCCTACAGTTACACCCCCTCTCCCTCCTCCTTCCCTCCATAACTCCATTATT
  consensus/80%                                CTGGATTCTCGTTCCACTTTGCCGCTCTGCCTACAGTTACACCCCCTCTCCCTCCTCCTTCCCTCCATAACTCCATTATT
  consensus/70%                                CTGGATTCTCGTTCCACTTTGCCGCTCTGCCTACAGTTACACCCCCTCTCCCTCCTCCTTCCCTCCATAACTCCATTATT

                                 cov    pid  81           .         1         .         .         .         .         :         . 160
1 FvH4_6g45860              100.0% 100.0%      ACCTCCAAAACTTTCAAAGACTTGAGAGTGAGGAGGAAACAACATAACAACATGTTGAAAGAAGAGAGCAATGGCGCCGC
2 gene04172-v1.0-hybrid     100.0% 100.0%      ACCTCCAAAACTTTCAAAGACTTGAGAGTGAGGAGGAAACAACATAACAACATGTTGAAAGAAGAGAGCAATGGCGCCGC
  consensus/100%                               ACCTCCAAAACTTTCAAAGACTTGAGAGTGAGGAGGAAACAACATAACAACATGTTGAAAGAAGAGAGCAATGGCGCCGC
  consensus/90%                                ACCTCCAAAACTTTCAAAGACTTGAGAGTGAGGAGGAAACAACATAACAACATGTTGAAAGAAGAGAGCAATGGCGCCGC
  consensus/80%                                ACCTCCAAAACTTTCAAAGACTTGAGAGTGAGGAGGAAACAACATAACAACATGTTGAAAGAAGAGAGCAATGGCGCCGC
  consensus/70%                                ACCTCCAAAACTTTCAAAGACTTGAGAGTGAGGAGGAAACAACATAACAACATGTTGAAAGAAGAGAGCAATGGCGCCGC

                                 cov    pid 161           .         .         .         2         .         .         .         . 240
1 FvH4_6g45860              100.0% 100.0%      GGCCGCGAACAGCTGGGCACGTGTCTGCGACACGTGCCGCTCCGCGCCCTGCACGGTCTACTGCCGTGCAGACTCCGCGT
2 gene04172-v1.0-hybrid     100.0% 100.0%      GGCCGCGAACAGCTGGGCACGTGTCTGCGACACGTGCCGCTCCGCGCCCTGCACGGTCTACTGCCGTGCAGACTCCGCGT
  consensus/100%                               GGCCGCGAACAGCTGGGCACGTGTCTGCGACACGTGCCGCTCCGCGCCCTGCACGGTCTACTGCCGTGCAGACTCCGCGT
  consensus/90%                                GGCCGCGAACAGCTGGGCACGTGTCTGCGACACGTGCCGCTCCGCGCCCTGCACGGTCTACTGCCGTGCAGACTCCGCGT
  consensus/80%                                GGCCGCGAACAGCTGGGCACGTGTCTGCGACACGTGCCGCTCCGCGCCCTGCACGGTCTACTGCCGTGCAGACTCCGCGT
  consensus/70%                                GGCCGCGAACAGCTGGGCACGTGTCTGCGACACGTGCCGCTCCGCGCCCTGCACGGTCTACTGCCGTGCAGACTCCGCGT

                                 cov    pid 241           :         .         .         .         .         3         .         . 320
1 FvH4_6g45860              100.0% 100.0%      ACCTCTGTTCCGGCTGCGACGCCACCATCCACGCGGCCAACCGCGTGGCGTCGCGCCACGAGCGCGTGTGGGTCTGCGAG
2 gene04172-v1.0-hybrid     100.0% 100.0%      ACCTCTGTTCCGGCTGCGACGCCACCATCCACGCGGCCAACCGCGTGGCGTCGCGCCACGAGCGCGTGTGGGTCTGCGAG
  consensus/100%                               ACCTCTGTTCCGGCTGCGACGCCACCATCCACGCGGCCAACCGCGTGGCGTCGCGCCACGAGCGCGTGTGGGTCTGCGAG
  consensus/90%                                ACCTCTGTTCCGGCTGCGACGCCACCATCCACGCGGCCAACCGCGTGGCGTCGCGCCACGAGCGCGTGTGGGTCTGCGAG
  consensus/80%                                ACCTCTGTTCCGGCTGCGACGCCACCATCCACGCGGCCAACCGCGTGGCGTCGCGCCACGAGCGCGTGTGGGTCTGCGAG
  consensus/70%                                ACCTCTGTTCCGGCTGCGACGCCACCATCCACGCGGCCAACCGCGTGGCGTCGCGCCACGAGCGCGTGTGGGTCTGCGAG

                                 cov    pid 321           .         .         :         .         .         .         .         4 400
1 FvH4_6g45860              100.0% 100.0%      GCCTGCGAGCGTGCGCCGGCCGCTTTACTCTGCAAGGCCGACGCGGCGTCGCTCTGCACGGCCTGCGACGCAGACATCCA
2 gene04172-v1.0-hybrid     100.0% 100.0%      GCCTGCGAGCGTGCGCCGGCCGCTTTACTCTGCAAGGCCGACGCGGCGTCGCTCTGCACGGCCTGCGACGCAGACATCCA
  consensus/100%                               GCCTGCGAGCGTGCGCCGGCCGCTTTACTCTGCAAGGCCGACGCGGCGTCGCTCTGCACGGCCTGCGACGCAGACATCCA
  consensus/90%                                GCCTGCGAGCGTGCGCCGGCCGCTTTACTCTGCAAGGCCGACGCGGCGTCGCTCTGCACGGCCTGCGACGCAGACATCCA
  consensus/80%                                GCCTGCGAGCGTGCGCCGGCCGCTTTACTCTGCAAGGCCGACGCGGCGTCGCTCTGCACGGCCTGCGACGCAGACATCCA
  consensus/70%                                GCCTGCGAGCGTGCGCCGGCCGCTTTACTCTGCAAGGCCGACGCGGCGTCGCTCTGCACGGCCTGCGACGCAGACATCCA

                                 cov    pid 401           .         .         .         .         :         .         .         . 480
1 FvH4_6g45860              100.0% 100.0%      CTCCGCCAACCCTCTGGCGCGTCGCCACCAGCGCGTCCCCATCCTCCCCATCTCCGGCGGTCAGATAGTGGTGGGGTCCA
2 gene04172-v1.0-hybrid     100.0% 100.0%      CTCCGCCAACCCTCTGGCGCGTCGCCACCAGCGCGTCCCCATCCTCCCCATCTCCGGCGGTCAGATAGTGGTGGGGTCCA
  consensus/100%                               CTCCGCCAACCCTCTGGCGCGTCGCCACCAGCGCGTCCCCATCCTCCCCATCTCCGGCGGTCAGATAGTGGTGGGGTCCA
  consensus/90%                                CTCCGCCAACCCTCTGGCGCGTCGCCACCAGCGCGTCCCCATCCTCCCCATCTCCGGCGGTCAGATAGTGGTGGGGTCCA
  consensus/80%                                CTCCGCCAACCCTCTGGCGCGTCGCCACCAGCGCGTCCCCATCCTCCCCATCTCCGGCGGTCAGATAGTGGTGGGGTCCA
  consensus/70%                                CTCCGCCAACCCTCTGGCGCGTCGCCACCAGCGCGTCCCCATCCTCCCCATCTCCGGCGGTCAGATAGTGGTGGGGTCCA

                                 cov    pid 481           .         5         .         .         .         .         :         . 560
1 FvH4_6g45860              100.0% 100.0%      CTCCGGCGGATACAACCGAGGACGGGTTCCTGAGCCAGGAAGGAGACGAGGAGGCCATGGATGAAGAAGACGAAGACGAG
2 gene04172-v1.0-hybrid     100.0% 100.0%      CTCCGGCGGATACAACCGAGGACGGGTTCCTGAGCCAGGAAGGAGACGAGGAGGCCATGGATGAAGAAGACGAAGACGAG
  consensus/100%                               CTCCGGCGGATACAACCGAGGACGGGTTCCTGAGCCAGGAAGGAGACGAGGAGGCCATGGATGAAGAAGACGAAGACGAG
  consensus/90%                                CTCCGGCGGATACAACCGAGGACGGGTTCCTGAGCCAGGAAGGAGACGAGGAGGCCATGGATGAAGAAGACGAAGACGAG
  consensus/80%                                CTCCGGCGGATACAACCGAGGACGGGTTCCTGAGCCAGGAAGGAGACGAGGAGGCCATGGATGAAGAAGACGAAGACGAG
  consensus/70%                                CTCCGGCGGATACAACCGAGGACGGGTTCCTGAGCCAGGAAGGAGACGAGGAGGCCATGGATGAAGAAGACGAAGACGAG

                                 cov    pid 561           .         .         .         6         .         .         .         . 640
1 FvH4_6g45860              100.0% 100.0%      GCTGCTTCCTGGCTGCTGCTGAATCCTGTGAAGAACAGCAATAGCCACAACAGTAACAACAACAACAATCCGAACAGTAA
2 gene04172-v1.0-hybrid     100.0% 100.0%      GCTGCTTCCTGGCTGCTGCTGAATCCTGTGAAGAACAGCAATAGCCACAACAGTAACAACAACAACAATCCGAACAGTAA
  consensus/100%                               GCTGCTTCCTGGCTGCTGCTGAATCCTGTGAAGAACAGCAATAGCCACAACAGTAACAACAACAACAATCCGAACAGTAA
  consensus/90%                                GCTGCTTCCTGGCTGCTGCTGAATCCTGTGAAGAACAGCAATAGCCACAACAGTAACAACAACAACAATCCGAACAGTAA
  consensus/80%                                GCTGCTTCCTGGCTGCTGCTGAATCCTGTGAAGAACAGCAATAGCCACAACAGTAACAACAACAACAATCCGAACAGTAA
  consensus/70%                                GCTGCTTCCTGGCTGCTGCTGAATCCTGTGAAGAACAGCAATAGCCACAACAGTAACAACAACAACAATCCGAACAGTAA

                                 cov    pid 641           :         .         .         .         .         7         .         . 720
1 FvH4_6g45860              100.0% 100.0%      CAACAACGGATTCTTCTTCGGAGTGGAGGTTGATGAGTACTTGGACCTTGTGGAGTACAACTCATCTGATCAGAACCAGT
2 gene04172-v1.0-hybrid     100.0% 100.0%      CAACAACGGATTCTTCTTCGGAGTGGAGGTTGATGAGTACTTGGACCTTGTGGAGTACAACTCATCTGATCAGAACCAGT
  consensus/100%                               CAACAACGGATTCTTCTTCGGAGTGGAGGTTGATGAGTACTTGGACCTTGTGGAGTACAACTCATCTGATCAGAACCAGT
  consensus/90%                                CAACAACGGATTCTTCTTCGGAGTGGAGGTTGATGAGTACTTGGACCTTGTGGAGTACAACTCATCTGATCAGAACCAGT
  consensus/80%                                CAACAACGGATTCTTCTTCGGAGTGGAGGTTGATGAGTACTTGGACCTTGTGGAGTACAACTCATCTGATCAGAACCAGT
  consensus/70%                                CAACAACGGATTCTTCTTCGGAGTGGAGGTTGATGAGTACTTGGACCTTGTGGAGTACAACTCATCTGATCAGAACCAGT

                                 cov    pid 721           .         .         :         .         .         .         .         8 800
1 FvH4_6g45860              100.0% 100.0%      TCAGTGGTACTACTGCTACTAATGACCAGCATAGCTATGGTGTGCCGCACAAGATCAGTTATGGAGGTGATAGTGTTGTA
2 gene04172-v1.0-hybrid     100.0% 100.0%      TCAGTGGTACTACTGCTACTAATGACCAGCATAGCTATGGTGTGCCGCACAAGATCAGTTATGGAGGTGATAGTGTTGTA
  consensus/100%                               TCAGTGGTACTACTGCTACTAATGACCAGCATAGCTATGGTGTGCCGCACAAGATCAGTTATGGAGGTGATAGTGTTGTA
  consensus/90%                                TCAGTGGTACTACTGCTACTAATGACCAGCATAGCTATGGTGTGCCGCACAAGATCAGTTATGGAGGTGATAGTGTTGTA
  consensus/80%                                TCAGTGGTACTACTGCTACTAATGACCAGCATAGCTATGGTGTGCCGCACAAGATCAGTTATGGAGGTGATAGTGTTGTA
  consensus/70%                                TCAGTGGTACTACTGCTACTAATGACCAGCATAGCTATGGTGTGCCGCACAAGATCAGTTATGGAGGTGATAGTGTTGTA

                                 cov    pid 801           .         .         .         .         :         .         .         . 880
1 FvH4_6g45860              100.0% 100.0%      CCGGTTCAGTATGGAGAAGGTAAAGTGACCCAGATGCAGATGCAGCAGAAGCATAATTTTCATCAGTTGGGGATGGAATA
```

```
2 gene04172-v1.0-hybrid 100.0% 100.0%    CCGGTTCAGTATGGAGAAGGTAAAGTGACCCAGATGCAGATGCAGCAGAAGCATAATTTTCATCAGTTGGGGATGGAATA
  consensus/100%                         CCGGTTCAGTATGGAGAAGGTAAAGTGACCCAGATGCAGATGCAGCAGAAGCATAATTTTCATCAGTTGGGGATGGAATA
  consensus/90%                          CCGGTTCAGTATGGAGAAGGTAAAGTGACCCAGATGCAGATGCAGCAGAAGCATAATTTTCATCAGTTGGGGATGGAATA
  consensus/80%                          CCGGTTCAGTATGGAGAAGGTAAAGTGACCCAGATGCAGATGCAGCAGAAGCATAATTTTCATCAGTTGGGGATGGAATA
  consensus/70%                          CCGGTTCAGTATGGAGAAGGTAAAGTGACCCAGATGCAGATGCAGCAGAAGCATAATTTTCATCAGTTGGGGATGGAATA

                           cov    pid  881       .         9         .         .         .         .         :         . 960
1 FvH4_6g45860            100.0% 100.0%    TGAGTCCTCAAAAGCTGCTTATGGTTATGATGGTTCCATTTCTCACACTGTAAGCTTCTAATTTCCACTTCATAATGCTT
2 gene04172-v1.0-hybrid 100.0% 100.0%    TGAGTCCTCAAAAGCTGCTTATGGTTATGATGGTTCCATTTCTCACACTGTAAGCTTCTAATTTCCACTTCATAATGCTT
  consensus/100%                         TGAGTCCTCAAAAGCTGCTTATGGTTATGATGGTTCCATTTCTCACACTGTAAGCTTCTAATTTCCACTTCATAATGCTT
  consensus/90%                          TGAGTCCTCAAAAGCTGCTTATGGTTATGATGGTTCCATTTCTCACACTGTAAGCTTCTAATTTCCACTTCATAATGCTT
  consensus/80%                          TGAGTCCTCAAAAGCTGCTTATGGTTATGATGGTTCCATTTCTCACACTGTAAGCTTCTAATTTCCACTTCATAATGCTT
  consensus/70%                          TGAGTCCTCAAAAGCTGCTTATGGTTATGATGGTTCCATTTCTCACACTGTAAGCTTCTAATTTCCACTTCATAATGCTT

                           cov    pid  961       .         .         .         0         .         .         .         . 1040
1 FvH4_6g45860            100.0% 100.0%    CTACGATTGGTTAATTACTTGTTTATTGCTAGCTTTTGGTTGATTAGCTATTGCGATTCCTAGTGAATAGACGTTTATCT
2 gene04172-v1.0-hybrid 100.0% 100.0%    CTACGATTGGTTAATTACTTGTTTATTGCTAGCTTTTGGTTGATTAGCTATTGCGATTCCTAGTGAATAGACGTTTATCT
  consensus/100%                         CTACGATTGGTTAATTACTTGTTTATTGCTAGCTTTTGGTTGATTAGCTATTGCGATTCCTAGTGAATAGACGTTTATCT
  consensus/90%                          CTACGATTGGTTAATTACTTGTTTATTGCTAGCTTTTGGTTGATTAGCTATTGCGATTCCTAGTGAATAGACGTTTATCT
  consensus/80%                          CTACGATTGGTTAATTACTTGTTTATTGCTAGCTTTTGGTTGATTAGCTATTGCGATTCCTAGTGAATAGACGTTTATCT
  consensus/70%                          CTACGATTGGTTAATTACTTGTTTATTGCTAGCTTTTGGTTGATTAGCTATTGCGATTCCTAGTGAATAGACGTTTATCT

                           cov    pid 1041       :         .         .         .         .         1         .         . 1120
1 FvH4_6g45860            100.0% 100.0%    GAGATGATGTTTTACTGGTGTAAAGATAAGCTTTCTTTTACTGGTAAAGCTAAAAAGAGAAAGCTAGCCTTCTACTGTTA
2 gene04172-v1.0-hybrid 100.0% 100.0%    GAGATGATGTTTTACTGGTGTAAAGATAAGCTTTCTTTTACTGGTAAAGCTAAAAAGAGAAAGCTAGCCTTCTACTGTTA
  consensus/100%                         GAGATGATGTTTTACTGGTGTAAAGATAAGCTTTCTTTTACTGGTAAAGCTAAAAAGAGAAAGCTAGCCTTCTACTGTTA
  consensus/90%                          GAGATGATGTTTTACTGGTGTAAAGATAAGCTTTCTTTTACTGGTAAAGCTAAAAAGAGAAAGCTAGCCTTCTACTGTTA
  consensus/80%                          GAGATGATGTTTTACTGGTGTAAAGATAAGCTTTCTTTTACTGGTAAAGCTAAAAAGAGAAAGCTAGCCTTCTACTGTTA
  consensus/70%                          GAGATGATGTTTTACTGGTGTAAAGATAAGCTTTCTTTTACTGGTAAAGCTAAAAAGAGAAAGCTAGCCTTCTACTGTTA

                           cov    pid 1121       .         .         :         .         .         .         .         2 1200
1 FvH4_6g45860            100.0% 100.0%    TCTGCACTTTTTTAGCTCTTTTGTCTCCAAATCTTTGCTTTAGTGGCAAAGAATATGAATGTTTCTATTTTGCCTTTCTC
2 gene04172-v1.0-hybrid 100.0% 100.0%    TCTGCACTTTTTTAGCTCTTTTGTCTCCAAATCTTTGCTTTAGTGGCAAAGAATATGAATGTTTCTATTTTGCCTTTCTC
  consensus/100%                         TCTGCACTTTTTTAGCTCTTTTGTCTCCAAATCTTTGCTTTAGTGGCAAAGAATATGAATGTTTCTATTTTGCCTTTCTC
  consensus/90%                          TCTGCACTTTTTTAGCTCTTTTGTCTCCAAATCTTTGCTTTAGTGGCAAAGAATATGAATGTTTCTATTTTGCCTTTCTC
  consensus/80%                          TCTGCACTTTTTTAGCTCTTTTGTCTCCAAATCTTTGCTTTAGTGGCAAAGAATATGAATGTTTCTATTTTGCCTTTCTC
  consensus/70%                          TCTGCACTTTTTTAGCTCTTTTGTCTCCAAATCTTTGCTTTAGTGGCAAAGAATATGAATGTTTCTATTTTGCCTTTCTC

                           cov    pid 1201       .         .         .         .         :         .         .         . 1280
1 FvH4_6g45860            100.0% 100.0%    CTTTTGCTTGCCCCTTTTGATCCAAGTAAGAAATTGACAGTCACCAAAGTGTGCAGTGTTACTTATCCTAGGTATATATT
2 gene04172-v1.0-hybrid 100.0% 100.0%    CTTTTGCTTGCCCCTTTTGATCCAAGTAAGAAATTGACAGTCACCAAAGTGTGCAGTGTTACTTATCCTAGGTATATATT
  consensus/100%                         CTTTTGCTTGCCCCTTTTGATCCAAGTAAGAAATTGACAGTCACCAAAGTGTGCAGTGTTACTTATCCTAGGTATATATT
  consensus/90%                          CTTTTGCTTGCCCCTTTTGATCCAAGTAAGAAATTGACAGTCACCAAAGTGTGCAGTGTTACTTATCCTAGGTATATATT
  consensus/80%                          CTTTTGCTTGCCCCTTTTGATCCAAGTAAGAAATTGACAGTCACCAAAGTGTGCAGTGTTACTTATCCTAGGTATATATT
  consensus/70%                          CTTTTGCTTGCCCCTTTTGATCCAAGTAAGAAATTGACAGTCACCAAAGTGTGCAGTGTTACTTATCCTAGGTATATATT

                           cov    pid 1281       .         3         .         .         .         .         :         . 1360
1 FvH4_6g45860            100.0% 100.0%    GTGATCTGAAGTGTTACTTTCTCTTGTGTAGTTCTGTACAGATACTTCAATAAGTCAGAGAATGATTTAGGAACATTTTG
2 gene04172-v1.0-hybrid 100.0% 100.0%    GTGATCTGAAGTGTTACTTTCTCTTGTGTAGTTCTGTACAGATACTTCAATAAGTCAGAGAATGATTTAGGAACATTTTG
  consensus/100%                         GTGATCTGAAGTGTTACTTTCTCTTGTGTAGTTCTGTACAGATACTTCAATAAGTCAGAGAATGATTTAGGAACATTTTG
  consensus/90%                          GTGATCTGAAGTGTTACTTTCTCTTGTGTAGTTCTGTACAGATACTTCAATAAGTCAGAGAATGATTTAGGAACATTTTG
  consensus/80%                          GTGATCTGAAGTGTTACTTTCTCTTGTGTAGTTCTGTACAGATACTTCAATAAGTCAGAGAATGATTTAGGAACATTTTG
  consensus/70%                          GTGATCTGAAGTGTTACTTTCTCTTGTGTAGTTCTGTACAGATACTTCAATAAGTCAGAGAATGATTTAGGAACATTTTG

                           cov    pid 1361       .         .         .         4         .         .         .         . 1440
1 FvH4_6g45860            100.0% 100.0%    AAAGGCATACGAGTTTTAGCTCTCCAATTCATTTCATTTTAATGGGGCTTATGCTTATTCAGGGTGTAAGCTACTCAACT
2 gene04172-v1.0-hybrid 100.0% 100.0%    AAAGGCATACGAGTTTTAGCTCTCCAATTCATTTCATTTTAATGGGGCTTATGCTTATTCAGGGTGTAAGCTACTCAACT
  consensus/100%                         AAAGGCATACGAGTTTTAGCTCTCCAATTCATTTCATTTTAATGGGGCTTATGCTTATTCAGGGTGTAAGCTACTCAACT
  consensus/90%                          AAAGGCATACGAGTTTTAGCTCTCCAATTCATTTCATTTTAATGGGGCTTATGCTTATTCAGGGTGTAAGCTACTCAACT
  consensus/80%                          AAAGGCATACGAGTTTTAGCTCTCCAATTCATTTCATTTTAATGGGGCTTATGCTTATTCAGGGTGTAAGCTACTCAACT
  consensus/70%                          AAAGGCATACGAGTTTTAGCTCTCCAATTCATTTCATTTTAATGGGGCTTATGCTTATTCAGGGTGTAAGCTACTCAACT

                           cov    pid 1441       :         .         .         .         .         5         .         . 1520
1 FvH4_6g45860            100.0% 100.0%    AGCTTATGTGGTGGAAGAGTGCATGAAAAGTCTAGAAAACCCCCAAACATAGTGAACAATCCCAAAAGCAAGTGTTGAAT
2 gene04172-v1.0-hybrid 100.0% 100.0%    AGCTTATGTGGTGGAAGAGTGCATGAAAAGTCTAGAAAACCCCCAAACATAGTGAACAATCCCAAAAGCAAGTGTTGAAT
  consensus/100%                         AGCTTATGTGGTGGAAGAGTGCATGAAAAGTCTAGAAAACCCCCAAACATAGTGAACAATCCCAAAAGCAAGTGTTGAAT
  consensus/90%                          AGCTTATGTGGTGGAAGAGTGCATGAAAAGTCTAGAAAACCCCCAAACATAGTGAACAATCCCAAAAGCAAGTGTTGAAT
  consensus/80%                          AGCTTATGTGGTGGAAGAGTGCATGAAAAGTCTAGAAAACCCCCAAACATAGTGAACAATCCCAAAAGCAAGTGTTGAAT
  consensus/70%                          AGCTTATGTGGTGGAAGAGTGCATGAAAAGTCTAGAAAACCCCCAAACATAGTGAACAATCCCAAAAGCAAGTGTTGAAT

                           cov    pid 1521       .         .         :         .         .         .         .         6 1600
1 FvH4_6g45860            100.0% 100.0%    TCATGCATCCTTTATAAATGATGAAAATATGAAATTATATTTGGTTCATCTTGACAGATGCTTTGATCTGCTAGCAATTT
2 gene04172-v1.0-hybrid 100.0% 100.0%    TCATGCATCCTTTATAAATGATGAAAATATGAAATTATATTTGGTTCATCTTGACAGATGCTTTGATCTGCTAGCAATTT
  consensus/100%                         TCATGCATCCTTTATAAATGATGAAAATATGAAATTATATTTGGTTCATCTTGACAGATGCTTTGATCTGCTAGCAATTT
  consensus/90%                          TCATGCATCCTTTATAAATGATGAAAATATGAAATTATATTTGGTTCATCTTGACAGATGCTTTGATCTGCTAGCAATTT
  consensus/80%                          TCATGCATCCTTTATAAATGATGAAAATATGAAATTATATTTGGTTCATCTTGACAGATGCTTTGATCTGCTAGCAATTT
  consensus/70%                          TCATGCATCCTTTATAAATGATGAAAATATGAAATTATATTTGGTTCATCTTGACAGATGCTTTGATCTGCTAGCAATTT

                           cov    pid 1601       .         .         .         .         :         .         .         . 1680
1 FvH4_6g45860            100.0% 100.0%    TTCCTGATGGTGCTTCTCTTTGATCATTTGATAGGTTTCTGTTTCATCCATGGATGTTGGAGTTGTACCAGACTCAACAA
2 gene04172-v1.0-hybrid 100.0% 100.0%    TTCCTGATGGTGCTTCTCTTTGATCATTTGATAGGTTTCTGTTTCATCCATGGATGTTGGAGTTGTACCAGACTCAACAA
  consensus/100%                         TTCCTGATGGTGCTTCTCTTTGATCATTTGATAGGTTTCTGTTTCATCCATGGATGTTGGAGTTGTACCAGACTCAACAA
  consensus/90%                          TTCCTGATGGTGCTTCTCTTTGATCATTTGATAGGTTTCTGTTTCATCCATGGATGTTGGAGTTGTACCAGACTCAACAA
  consensus/80%                          TTCCTGATGGTGCTTCTCTTTGATCATTTGATAGGTTTCTGTTTCATCCATGGATGTTGGAGTTGTACCAGACTCAACAA
  consensus/70%                          TTCCTGATGGTGCTTCTCTTTGATCATTTGATAGGTTTCTGTTTCATCCATGGATGTTGGAGTTGTACCAGACTCAACAA

                           cov    pid 1681       .         7         .         .         .         .         :         . 1760
1 FvH4_6g45860            100.0% 100.0%    TGAGTGAAATGTCTGTCTGTCACCCAAGAACACCGAAAGGAACAATAGACCTTTTTAATGGACCTACAATTCAGATTCCA
2 gene04172-v1.0-hybrid 100.0% 100.0%    TGAGTGAAATGTCTGTCTGTCACCCAAGAACACCGAAAGGAACAATAGACCTTTTTAATGGACCTACAATTCAGATTCCA
```

```
  consensus/100%                                 TGAGTGAAATGTCTGTCTGTCACCCAAGAACACCGAAAGGAACAATAGACCTTTTTAATGGACCTACAATTCAGATTCCA
  consensus/90%                                  TGAGTGAAATGTCTGTCTGTCACCCAAGAACACCGAAAGGAACAATAGACCTTTTTAATGGACCTACAATTCAGATTCCA
  consensus/80%                                  TGAGTGAAATGTCTGTCTGTCACCCAAGAACACCGAAAGGAACAATAGACCTTTTTAATGGACCTACAATTCAGATTCCA
  consensus/70%                                  TGAGTGAAATGTCTGTCTGTCACCCAAGAACACCGAAAGGAACAATAGACCTTTTTAATGGACCTACAATTCAGATTCCA

                          cov    pid 1761                 .         .         .         8         .         .         .         . 1840
1 FvH4_6g45860           100.0% 100.0%       ACCCAACTAAGTCCTATGGACAGGGAGGCCAGGGTCCTCAGATACAGAGAGAAAAAGAAGACGAGGAAGTTTGAGAAAAC
2 gene04172-v1.0-hybrid  100.0% 100.0%       ACCCAACTAAGTCCTATGGACAGGGAGGCCAGGGTCCTCAGATACAGAGAGAAAAAGAAGACGAGGAAGTTTGAGAAAAC
  consensus/100%                             ACCCAACTAAGTCCTATGGACAGGGAGGCCAGGGTCCTCAGATACAGAGAGAAAAAGAAGACGAGGAAGTTTGAGAAAAC
  consensus/90%                              ACCCAACTAAGTCCTATGGACAGGGAGGCCAGGGTCCTCAGATACAGAGAGAAAAAGAAGACGAGGAAGTTTGAGAAAAC
  consensus/80%                              ACCCAACTAAGTCCTATGGACAGGGAGGCCAGGGTCCTCAGATACAGAGAGAAAAAGAAGACGAGGAAGTTTGAGAAAAC
  consensus/70%                              ACCCAACTAAGTCCTATGGACAGGGAGGCCAGGGTCCTCAGATACAGAGAGAAAAAGAAGACGAGGAAGTTTGAGAAAAC

                          cov    pid 1841                 :         .         .         .         .         9         .         . 1920
1 FvH4_6g45860           100.0% 100.0%       AATCCGGTATGCCTCAAGGAAGGCCTATGCAGAGACTAGACCCCGGATCAAGGGCCGGTTTGCAAAGCGAACAGACATCG
2 gene04172-v1.0-hybrid  100.0% 100.0%       AATCCGGTATGCCTCAAGGAAGGCCTATGCAGAGACTAGACCCCGGATCAAGGGCCGGTTTGCAAAGCGAACAGACATCG
  consensus/100%                             AATCCGGTATGCCTCAAGGAAGGCCTATGCAGAGACTAGACCCCGGATCAAGGGCCGGTTTGCAAAGCGAACAGACATCG
  consensus/90%                              AATCCGGTATGCCTCAAGGAAGGCCTATGCAGAGACTAGACCCCGGATCAAGGGCCGGTTTGCAAAGCGAACAGACATCG
  consensus/80%                              AATCCGGTATGCCTCAAGGAAGGCCTATGCAGAGACTAGACCCCGGATCAAGGGCCGGTTTGCAAAGCGAACAGACATCG
  consensus/70%                              AATCCGGTATGCCTCAAGGAAGGCCTATGCAGAGACTAGACCCCGGATCAAGGGCCGGTTTGCAAAGCGAACAGACATCG

                          cov    pid 1921                 .         .         :         .         .         .         ] 1994
1 FvH4_6g45860           100.0% 100.0%       ACGTTGAAGTGGATCAGATGTTCTCCACATCACTTATGGGAGAAACTGGATACGGCATTGTTCCTTCATACTAA
2 gene04172-v1.0-hybrid  100.0% 100.0%       ACGTTGAAGTGGATCAGATGTTCTCCACATCACTTATGGGAGAAACTGGATACGGCATTGTTCCTTCATACTAA
  consensus/100%                             ACGTTGAAGTGGATCAGATGTTCTCCACATCACTTATGGGAGAAACTGGATACGGCATTGTTCCTTCATACTAA
  consensus/90%                              ACGTTGAAGTGGATCAGATGTTCTCCACATCACTTATGGGAGAAACTGGATACGGCATTGTTCCTTCATACTAA
  consensus/80%                              ACGTTGAAGTGGATCAGATGTTCTCCACATCACTTATGGGAGAAACTGGATACGGCATTGTTCCTTCATACTAA
  consensus/70%                              ACGTTGAAGTGGATCAGATGTTCTCCACATCACTTATGGGAGAAACTGGATACGGCATTGTTCCTTCATACTAA
```

MView 1.63, Copyright © 1997–2018 Nigel P. Brown
