# Supplementary material for: An Evolutionary Analysis of B-Box Transcription Factors in Strawberry Reveals the Role of FaBBx28c1 in the Regulation of Flowering Time
Source: Int J Mol Sci. 2021 Oct 29;22(21):11766. doi: 10.3390/ijms222111766 (PMC8583817; doi:10.3390/ijms222111766)
Supplement: Supplementary file 1 [file ijms-22-11766-s001.zip › SFiles/FileS.pdf]

FileS1

1. The FaBBX28c1 overexpression vector
>BBX28c1 OverExpression Vector
gatctgagggtaaatttctagtttttctccttcattttcttggttaggacccttttctctttttatttttttgagctttg
atctttctttaaactgatctatttttaattgattggttatggtgtaaatattacatagctttaactgataatctgatta
ctttatttcgtgtgtctatgatgatgatgatagttacagaaccgacgactcgtccgtcctgtagaaaccccaacccgtga
aatcaaaaaactcgacggcctgtgggcattcagtctggatcgcgaaaactgtggaattgatcagcgttggtgggaaagcg
cgttacaagaaagccgggcaattgctgtgccaggcagttttaacgatcagttcgccgatgcagatattcgtaattatgcg
ggcaacgtctggtatcagcgcgaagtctttataccgaaaggttgggcaggccagcgtatcgtgctgcgtttcgatgcggt
cactcattacggcaaagtgtgggtcaataatcaggaagtgatggagcatcagggcggctatacgccatttgaagccgatg
tcacgccgtatgttattgccgggaaaagtgtacgtatcaccgtttgtgtgaacaacgaactgaactggcagactatcccg
ccgggaatggtgattaccgacgaaaacggcaagaaaaagcagtcttacttccatgatttctttaactatgccggaatcca
tcgcagcgtaatgctctacaccacgccgaacacctgggtggacgatatcaccgtggtgacgcatgtcgcgcaagactgta
accacgcgtctgttgactggcaggtggtggccaatggtgatgtcagcgttgaactgcgtgatgcggatcaacaggtggtt
gcaactggacaaggcactagcgggactttgcaagtggtgaatccgcacctctggcaaccgggtgaaggttatctctatga
actcgaagtcacagccaaaagccagacagagtctgatatctacccgcttcgcgtcggcatccggtcagtggcagtgaagg
gccaacagttcctgattaaccacaaaccgttctactttactggctttggtcgtcatgaagatgcggacttacgtggcaaa
ggattcgataacgtgctgatggtgcacgaccacgcattaatggactggattggggccaactcctaccgtacctcgcatta
cccttacgctgaagagatgctcgactgggcagatgaacatggcatcgtggtgattgatgaaactgctgctgtcggctttc
agctgtctttaggcattggtttcgaagcgggcaacaagccgaaagaactgtacagcgaagaggcagtcaacggggaaact
cagcaagcgcacttacaggcgattaaagagctgatagcgcgtgacaaaaaccacccaagcgtggtgatgtggagtattgc
caacgaaccggatacccgtccgcaaggtgcacgggaatatttcgcgccactggcggaagcaacgcgtaaactcgacccga
cgcgtccgatcacctgcgtcaatgtaatgttctgcgacgctcacaccgataccatcagcgatctctttgatgtgctgtgc
ctgaaccgttattacggatggtatgtccaaagcggcgatttggaaacggcagagaaggtactggaaaaagaacttctggc
ctggcaggagaaactgcatcagccgattatcatcaccgaatacggcgtggatacgttagccgggctgcactcaatgtaca
ccgacatgtggagtgaagagtatcagtgtgcatggctggatatgtatcaccgcgtctttgatcgcgtcagcgccgtcgtc
ggtgaacaggtatggaatttcgccgattttgcgacctcgcaaggcatattgcgcgttggcggtaacaagaaagggatctt
cactcgcgaccgcaaaccgaagtcggcggcttttctgctgcaaaaacgctggactggcatgaacttcggtgaaaaaccgc
agcagggaggcaaacaagctagccaccaccaccaccaccacgtgtgaattacaggtgaccagctcgaatttccccgatcg
ttcaaacatttggcaataaagtttcttaagattgaatcctgttgccggtcttgcgatgattatcatataatttctgttga
attacgttaagcatgtaataattaacatgtaatgcatgacgttatttatgagatgggtttttatgattagagtcccgcaa
ttatacatttaatacgcgatagaaaacaaaatatagcgcgcaaactaggataaattatcgcgcgcggtgtcatctatgtt
actagatcgggaattaaactatcagtgtttgacaggatatattggcgggtaaacctaagagaaaagagcgtttattagaa
taacggatatttaaaagggcgtgaaaaggtttatccgttcgtccatttgtatgtgcatgccaaccacagggttcccctcg
ggatcaaagtactttgatccaacccctccgctgctatagtgcagtcggcttctgacgttcagtgcagccgtcttctgaaa
acgacatgtcgcacaagtcctaagttacgcgacaggctgccgccctgcccttttcctggcgttttcttgtcgcgtgtttt
agtcgcataaagtagaatacttgcgactagaaccggagacattacgccatgaacaagagcgccgccgctggcctgctggg
ctatgcccgcgtcagcaccgacgaccaggacttgaccaaccaacgggccgaactgcacgcggccggctgcaccaagctgt
tttccgagaagatcaccggcaccaggcgcgaccgcccggagctggccaggatgcttgaccacctacgccctggcgacgtt
gtgacagtgaccaggctagaccgcctggcccgcagcacccgcgacctactggacattgccgagcgcatccaggaggccgg
cgcgggcctgcgtagcctggcagagccgtgggccgacaccaccacgccggccggccgcatggtgttgaccgtgttcgccg
gcattgccgagttcgagcgttccctaatcatcgaccgcacccggagcgggcgcgaggccgccaaggcccgaggcgtgaag
tttggcccccgccctaccctcaccccggcacagatcgcgcacgcccgcgagctgatcgaccaggaaggccgcaccgtgaa
agaggcggctgcactgcttggcgtgcatcgctcgaccctgtaccgcgcacttgagcgcagcgaggaagtgacgcccaccg
aggccaggcggcgcggtgccttccgtgaggacgcattgaccgaggccgacgccctggcggccgccgagaatgaacgccaa
gaggaacaagcatgaaaccgcaccaggacggccaggacgaaccgtttttcattaccgaagagatcgaggcggagatgatc
gcggccgggtacgtgttcgagccgcccgcgcacgtctcaaccgtgcggctgcatgaaatcctggccggtttgtctgatgc
caagctggcggcctggccggccagcttggccgctgaagaaaccgagcgccgccgtctaaaaaggtgatgtgtatttgagt
aaaacagcttgcgtcatgcggtcgctgcgtatatgatgcgatgagtaaataaacaaatacgcaaggggaacgcatgaagg
ttatcgctgtacttaaccagaaaggcgggtcaggcaagacgaccatcgcaacccatctagcccgcgccctgcaactcgcc
ggggccgatgttctgttagtcgattccgatccccagggcagtgcccgcgattgggcggccgtgcgggaagatcaaccgct

aaccgttgtcggcatcgaccgcccgacgattgaccgcgacgtgaaggccatcggccggcgcgacttcgtagtgatcgacg
gagcgccccaggcggcggacttggctgtgtccgcgatcaaggcagccgacttcgtgctgattccggtgcagccaagccct
tacgacatatgggccaccgccgacctggtggagctggttaagcagcgcattgaggtcacggatggaaggctacaagcggc
ctttgtcgtgtcgcgggcgatcaaaggcacgcgcatcggcggtgaggttgccgaggcgctggccgggtacgagctgccca
ttcttgagtcccgtatcacgcagcgcgtgagctacccaggcactgccgccgccggcacaaccgttcttgaatcagaaccc
gagggcgacgctgcccgcgaggtccaggcgctggccgctgaaattaaatcaaaactcatttgagttaatgaggtaaagag
aaaatgagcaaaagcacaaacacgctaagtgccggccgtccgagcgcacgcagcagcaaggctgcaacgttggccagcct
ggcagacacgccagccatgaagcgggtcaactttcagttgccggcggaggatcacaccaagctgaagatgtacgcggtac
gccaaggcaagaccattaccgagctgctatctgaatacatcgcgcagctaccagagtaaatgagcaaatgaataaatgag
tagatgaattttagcggctaaaggaggcggcatggaaaatcaagaacaaccaggcaccgacgccgtggaatgccccatgt
gtggaggaacgggcggttggccaggcgtaagcggctgggttgtctgccggccctgcaatggcactggaacccccaagccc
gaggaatcggcgtgacggtcgcaaaccatccggcccggtacaaatcggcgcggcgctgggtgatgacctggtggagaagt
tgaaggccgcgcaggccgcccagcggcaacgcatcgaggcagaagcacgccccggtgaatcgtggcaagcggccgctgat
cgaatccgcaaagaatcccggcaaccgccggcagccggtgcgccgtcgattaggaagccgcccaagggcgacgagcaacc
agatttttcgttccgatgctctatgacgtgggcacccgcgatagtcgcagcatcatggacgtggccgttttccgtctgt
cgaagcgtgaccgacgagctggcgaggtgatccgctacgagcttccagacgggcacgtagaggtttccgcagggccggcc
ggcatggccagtgtgtgggattacgacctggtactgatggcggtttcccatctaaccgaatccatgaaccgataccggga
agggaagggagacaagcccggccgcgtgttccgtccacacgttgcggacgtactcaagttctgccggcgagccgatggcg
gaaagcagaaagacgacctggtagaaacctgcattcggttaaacaccacgcacgttgccatgcagcgtacgaagaaggcc
aagaacggccgcctggtgacggtatccgagggtgaagccttgattagccgctacaagatcgtaaagagcgaaaccgggcg
gccggagtacatcgagatcgagctagctgattggatgtaccgcgagatcacagaaggcaagaacccggacgtgctgacgg
ttcaccccgattactttttgatcgatcccggcatcggccgttttctctaccgcctggcacgccgcgccgcaggcaaggca
gaagccagatggttgttcaagacgatctacgaacgcagtggcagcgccggagagttcaagaagttctgtttcaccgtgcg
caagctgatcgggtcaaatgacctgccggagtacgatttgaaggaggaggcggggcaggctggcccgatcctagtcatgc
gctaccgcaacctgatcgagggcgaagcatccgccggttcctaatgtacggagcagatgctagggcaaattgccctagca
ggggaaaaaggtcgaaaaggtctctttcctgtggatagcacgtacattgggaacccaaagccgtacattgggaaccggaa
cccgtacattgggaacccaaagccgtacattgggaaccggtcacacatgtaagtgactgatataaaagagaaaaaaggcg
atttttccgcctaaaactctttaaaacttattaaaactcttaaaacccgcctggcctgtgcataactgtctggccagcgc
acagccgaagagctgcaaaaagcgcctacccttcggtcgctgcgctccctacgccccgccgcttcgcgtcggcctatcgc
ggccgctggccgctcaaaaatggctggcctacggccaggcaatctaccagggcgcggacaagccgcgccgtcgccactcg
accgccggcgcccacatcaaggcaccctgcctcgcgcgtttcggtgatgacggtgaaaacctctgacacatgcagctccc
ggagacggtcacagcttgtctgtaagcggatgccgggagcagacaagcccgtcagggcgcgtcagcgggtgttggcgggt
gtcggggcgcagccatgacccagtcacgtagcgatagcggagtgtatactggcttaactatgcggcatcagagcagattg
tactgagagtgcaccatatgcggtgtgaaataccgcacagatgcgtaaggagaaaataccgcatcaggcgctcttccgct
tcctcgctcactgactcgctgcgctcggtcgttcggctgcggcgagcggtatcagctcactcaaaggcggtaatacggtt
atccacagaatcaggggataacgcaggaaagaacatgtgagcaaaaggccagcaaaaggccaggaaccgtaaaaaggccg
cgttgctggcgtttttccataggctccgcccccctgacgagcatcacaaaaatcgacgctcaagtcagaggtggcgaaac
ccgacaggactataaagataccaggcgtttccccctggaagctccctcgtgcgctctcctgttccgaccctgccgcttac
cggatacctgtccgcctttctcccttcgggaagcgtggcgctttctcatagctcacgctgtaggtatctcagttcggtgt
aggtcgttcgctccaagctgggctgtgtgcacgaaccccccgttcagcccgaccgctgcgccttatccggtaactatcgt
cttgagtccaacccggtaagacacgacttatcgccactggcagcagccactggtaacaggattagcagagcgaggtatgt
aggcggtgctacagagttcttgaagtggtggcctaactacggctacactagaaggacagtatttggtatctgcgctctgc
tgaagccagttaccttcggaaaaagagttggtagctcttgatccggcaaacaaaccaccgctggtagcggtggtttttt
gtttgcaagcagcagattacgcgcagaaaaaaaggatctcaagaagatcctttgatcttttctacggggtctgacgctca
gtggaacgaaaactcacgttaagggattttggtcatgcattctaggtactaaaacaattcatccagtaaaatataatatt
ttattttctcccaatcaggcttgatccccagtaagtcaaaaaatagctcgacatactgttcttccccgatatcctccctg
atcgaccggacgcagaaggcaatgtcataccacttgtccgccctgccgcttctcccaagatcaataaagccacttacttt
gccatctttcacaaagatgttgctgtctcccaggtcgccgtgggaaaagacaagttcctcttcgggcttttccgtcttta
aaaaatcatacagctcgcgcggatctttaaatggagtgtcttcttcccagttttcgcaatccacatcggccagatcgtta
ttcagtaagtaatccaattcggctaagcggctgtctaagctattcgtatagggacaatccgatatgtcgatggagtgaaa
gagcctgatgcactccgcatacagctcgataatcttttcagggctttgttcatcttcatactcttccgagcaaaggacgc
catcggcctcactcatgagcagattgctccagccatcatgccgttcaaagtgcaggacctttggaacaggcagctttcct

tccagccatagcatcatgtccttttcccgttccacatcataggtggtccctttataccggctgtccgtcatttttaaata
taggttttcattttctcccaccagcttatataccttagcaggagacattccttccgtatcttttacgcagcggtattttt
cgatcagtttttcaattccggtgatattctcattttagccatttattatttccttcctcttttctacagtatttaaaga
taccccaagaagctaattataacaagacgaactccaattcactgttccttgcattctaaaaccttaaataccagaaaaca
gctttttcaaagttgttttcaaagttggcgtataacatagtatcgacggagccgattttgaaaccgcggtgatcacaggc
agcaacgctctgtcatcgttacaatcaacatgctaccctccgcgagatcatccgtgtttcaaacccggcagcttagttgc
cgttcttccgaatagcatcggtaacatgagcaaagtctgccgccttacaacggctctcccgctgacgccgtcccggactg
atgggctgcctgtatcgagtggtgattttgtgccgagctgccggtcggggagctgttggctggctggtggcaggatatat
tgtggtgtaaacaaattgacgcttagacaacttaataacacattgcggacgtttttaatgtactgaattaacgccgaatt
aattcgggggatctggattttagtactggattttggttttaggaattagaaattttattgatagaagtattttacaaata
caaatacatactaagggtttcttatatgctcaacacatgagcgaaaccctataggaaccctaattcccttatctgggaac
tactcacacattattatggagaaactcgagcttgtcgatcgacagatccggtcggcatctactctatttctttgccctcg
gacgagtgctggggcgtcggtttccactatcggcgagtacttctacacagccatcggtccagacggccgcgcttctgcgg
gcgatttgtgtacgcccgacagtcccggctccggatcggacgattgcgtcgcatcgaccctgcgcccaagctgcatcatc
gaaattgccgtcaaccaagctctgatagagttggtcaagaccaatgcggagcatatacgcccggagtcgtggcgatcctg
caagctccggatgcctccgctcgaagtagcgcgtctgctgctccatacaagccaaccacggcctccagaagaagatgttg
gcgacctcgtattgggaatccccgaacatcgcctcgctccagtcaatgaccgctgttatgcggccattgtccgtcaggac
attgttggagccgaaatccgcgtgcacgaggtgccggacttcggggcagtcctcggcccaaagcatcagctcatcgagag
cctgcgcgacggacgcactgacggtgtcgtccatcacagtttgccagtgatacacatggggatcagcaatcgcgcatatg
aaatcacgccatgtagtgtattgaccgattccttgcggtccgaatgggccgaacccgctcgtctggctaagatcggccgc
agcgatcgcatccatagcctccgcgaccggttgtagaacagcgggcagttcggtttcaggcaggtcttgcaacgtgacac
cctgtgcacggcgggagatgcaataggtcaggctctcgctaaactccccaatgtcaagcacttccggaatcgggagcgcg
gccgatgcaaagtgccgataaacataacgatctttgtagaaaccatcggcgcagctatttacccgcaggacatatccacg
ccctcctacatcgaagctgaaagcacgagattcttcgccctccgagagctgcatcaggtcggagacgctgtcgaactttt
cgatcagaaacttctcgacagacgtcgcggtgagttcaggctttttcatatctcattgccccccgggatctgcgaaagct
cgagagagatagatttgtagagagagactggtgatttcagcgtgtcctctccaaatgaaatgaacttccttatatagagg
aaggtcttgcgaaggatagtgggattgtgcgtcatcccttacgtcagtggagatatcacatcaatccacttgctttgaag
acgtggttggaacgtcttctttttccacgatgctcctcgtgggtgggggtccatctttgggaccactgtcggcagaggca
tcttgaacgatagcctttcctttatcgcaatgatggcatttgtaggtgccaccttccttttctactgtccttttgatgaa
gtgacagatagctgggcaatggaatccgaggaggtttcccgatattaccctttgttgaaaagtctcaatagccctttggt
cttctgagactgtatctttgatattcttggagtagacgagagtgtcgtgctccaccatgttatcacatcaatccacttgc
tttgaagacgtggttggaacgtcttctttttccacgatgctcctcgtgggtgggggtccatctttgggaccactgtcggc
agaggcatcttgaacgatagcctttcctttatcgcaatgatggcatttgtaggtgccaccttccttttctactgtccttt
tgatgaagtgacagatagctgggcaatggaatccgaggaggtttcccgatattaccctttgttgaaaagtctcaatagcc
ctttggtcttctgagactgtatctttgatattcttggagtagacgagagtgtcgtgctccaccatgttggcaagctgctc
tagccaatacgcaaaccgcctctccccgcgcgttggccgattcattaatgcagctggcacgacaggtttcccgactggaa
agcgggcagtgagcgcaacgcaattaatgtgagttagctcactcattaggcaccccaggctttacactttatgcttccgg
ctcgtatgttgtgtggaattgtgagcggataacaatttcacacaggaaacagctatgacATGATTACGAATTCTCATGTT
TGACAGCTTATCATCGGATCTAGTAACATAGATGACACCGCGCGCGATAATTTATCCTA
GTTTGCGCGCTATATTTTGTT
TTCTATCGCGTATTAAATGTATAATTGCGGGACTCTAATCATAAAAACCCATCTCATAA
ATAACGTCATGCATTACATGT
TAATTATTACATGCTTAACGTAATTCAACAGAAATTATATGATAATCATCGCAAGACCG
GCAACAGGATTCAATCTTAAG
AAACTTTATTGCCAAATGTTTGAACGATCTGCAGCCCGGGGGATCCTCATATCAGAGGGATA
CGAGGCTCGGAGGAGTCA
AGATCGACGGCTTCGCTGAGTTTACGGTCATTTTGCCGGAAATTTCTCCTCAGAGATTC
AATAGGGAGCGAGAATCTCGG
ACCTGAACCGTTAAGATCCAGTTTCCGGTCTTTTGAAGGTATCCTGCAGTCAATCGAAG
TTGCGCCGCCGTCGGTATGGG
CGGTGCAAGCTGTTTCACACCTCAGTCGTGCGGTTAAACGGCGGAGATCGTCATGAGA
GCGAAGATCAGAAGCATTATCT

CTGGTTCGCTTCAACGAAACCGCGCCGACTGAGCGGCGGCCATATCTATCGTCACTGTG
AGCCCCTTCGCTGCTCGAAGA
GCTCGCGGAGGGCGGAGGAGGCGTCGAAGCCCAAGGCACCACCTGATTCTCACCTTCC
TCATCGAAATCAAGATCGCCGT
CGTCGTCGTCAGAATCGGAGTCCTGATCTTCATCTAGGTCATCATCATCCGTATCATCA
TTACCGGCCTCGCTCTCTTCG
TCGTCGCCTCTGCTTTCGTTTCTGGCCACACACCGCTCGCAGACGGAAAAAGTATGGCC
GAGCTTCTCGCCGGAGGCTTT
CCAGGCCGTTGGCGCGTGGCACGCTTGGCAGAGGAGCGTCCTGGAGTGCCTGGCCACC
AGGAAATTGGCGCCGTGAACCT
TGAAATCGCAGTCCCAGCAGAGAATTGCCTGGTCAGATTCGCAGTAAGTCCGAGCAGG
TAGCTGGCAGAGCTCGCAGTTC
TTCATGGTACffcgggccccccctcgaggtcgacggtatcgataagcttgatAAACTAGAGTCCCCCGTGTTCTC
TCCAAAT
GAAATGAACTTCCTTATATAGAGGAAGGGTCTTGCGAAGGATAGTGGGATTGTGCGTC
ATCCCTTACGTCAGTGGAGATA
TCACATCAATCCACTTGCTTTGAAGACGTGGTTGGAACGTCTTCTTTTTCCACGATGCTC
CTCGTGGGTGGGGGTCCATC
TTTGGGACCACTGTCGGCAGAGGCATCTTCAACGATGGCCTTTCCTTTATCGCAATGAT
GGCATTTGTAGGAGCCACCTT
CCTTTTCCACTATCTTCACAATAAAGTGACAGATAGCTGGGCAATGGAATCCGAGGAG
GTTTCCGGATATTACCCTTTGT
TGAAAAGTCTCAATTGCCCTTTGGTCTTCTGAGACTGTATCTTTGATATTTTTGGAGTAG
ACAAGTGTGTCGTGCTCCAC
CATGTTGACGAAGATTTTCTTCTTGTCATTGAGTCGTAAGAGACTCTGTATGAACTGTT
CGCCAGTCTTTACGGCGAGTT
CTGTTAGGTCCTCTATTTGAATCTTTGACTCCATGGCCTTTGATTCAGTGGGAACTACCT
TTTTAGAGACTCCAATCTCT
ATTACTTGCCTTGGTTTGTGAAGCAAGCCTTGAATCGTCCATACTGGAATAGTACTTCT
GATCTTGAGAAATATATCTTT
CTCTGTGTTCTTGATGCAGTTAGTCCTGAATCTTTTGACTGCATCTTTAACCTTCTTGGG
AAGGTATTTGATTTCCTGGA
GATTATTGCTCGGGTAGATCGTCTTGATGAGACCTGCTGCGTAAGCCTCTCTAACCATC
TGTGGGTTAGCATTCTTTCTG
AAAtTGAAAAGGCtAATCTGGGgacctgcaggcatgcaagcttggcactggccgtcgttttacaacgtcgtgactgggaa
aaccctggcgttacccaacttaatcgccttgcagcacatccccctttcgccagctggcgtaatagcgaagaggcccgcac
cgatcgcccttcccaacagttgcgcagcctgaatggcgaatgctagagcagcttgagcttggatcagattgtcgtttccc
gccttcagtttagcttcatggagtcaaagattcaaatagaggacctaacagaactcgccgtaaagactggcgaacagttc
atacagagtctcttacgactcaatgacaagaagaaaatcttcgtcaacatggtggagcacgacacacttgtctactccaa
aaatatcaaagatacagtctcagaagaccaaagggcaattgagacttttcaacaaagggtaatatccggaaacctcctcg
gattccattgcccagctatctgtcactttattgtgaagatagtggaaaaggaaggtggctcctacaaatgccatcattgc
gataaaggaaaggccatcgttgaagatgcctctgccgacagtggtcccaaagatggacccccacccacgaggagcatcgt
ggaaaaagaagacgttccaaccacgtcttcaaagcaagtggattgatgtgatatctccactgacgtaagggatgacgcac
aatcccactatccttcgcaagacccttcctctatataaggaagttcatttcatttggagagaacacgggggactcttgac
catggta

Note : the ORF of FaBBX28c1 is marked by green color

2. The plasmid vecotr for subcellular localization analysis

>SubCellularVector_N_Fusion
cgtaatcatggtcatagctgtttcctgtgtgaaattgttatccgctcacaattccacacaacatacgagccggaagcata

aagtgtaaagcctggggtgcctaatgagtgagctaactcacattaattgcgttgcgctcactgcccgctttccagtcggg
aaacctgtcgtgccagctgcattaatgaatcggccaacgcgcggggagaggcggtttgcgtattggctagagcagcttgc
caacatggtggagcacgacactctcgtctactccaagaatatcaaagatacagtctcagaagaccaaagggctattgaga
cttttcaacaaagggtaatatcgggaaacctcctcggattccattgcccagctatctgtcacttcatcaaaaggacagta
gaaaaggaaggtggcacctacaaatgccatcattgcgataaaggaaaggctatcgttcaagatgcctctgccgacagtgg
tcccaaagatggacccccacccacgaggagcatcgtggaaaaagaagacgttccaaccacgtcttcaaagcaagtggatt
gatgtgataacatggtggagcacgacactctcgtctactccaagaatatcaaagatacagtctcagaagaccaaagggct
attgagacttttcaacaaagggtaatatcgggaaacctcctcggattccattgcccagctatctgtcacttcatcaaaag
gacagtagaaaaggaaggtggcacctacaaatgccatcattgcgataaaggaaaggctatcgttcaagatgcctctgccg
acagtggtcccaaagatggacccccacccacgaggagcatcgtggaaaaagaagacgttccaaccacgtcttcaaagcaa
gtggattgatgtgatatctccactgacgtaagggatgacgcacaatcccactatccttcgcaagaccttcctctatataa
ggaagttcatttcatttggagaggacacgctgaaatcaccagtctctctctacaaatctatctctctcgagctttcgcag
atcccgggggcaatgagatatgaaaaagcctgaactcaccgcgacgtctgtcgagaagtttctgatcgaaaagttcgac
agcgtctccgacctgatgcagctctcggagggcgaagaatctcgtgctttcagcttcgatgtaggagggcgtggatatgt
cctgcgggtaaatagctgcgccgatggtttctacaaagatcgttatgtttatcggcactttgcatcggccgcgctcccga
ttccggaagtgcttgacattggggagtttagcgagagcctgacctattgcatctcccgccgtgcacagggtgtcacgttg
caagacctgcctgaaaccgaactgcccgctgttctacaaccggtcgcggaggctatggatgcgatcgctgcggccgatct
tagccagacgagcgggttcggcccattcggaccgcaaggaatcggtcaatacactacatggcgtgatttcatatgcgcga
ttgctgatccccatgtgtatcactggcaaactgtgatggacgacaccgtcagtgcgtccgtcgcgcaggctctcgatgag
ctgatgctttgggccgaggactgccccgaagtccggcacctcgtgcacgcggatttcggctccaacaatgtcctgacgga
caatggccgcataacagcggtcattgactggagcgaggcgatgttcggggattcccaatacgaggtcgccaacatcttct
tctggaggccgtggttggcttgtatggagcagcagacgcgctacttcgagcggaggcatccggagcttgcaggatcgcca
cgactccgggcgtatatgctccgcattggtcttgaccaactctatcagagcttggttgacggcaatttcgatgatgcagc
ttgggcgcagggtcgatgcgacgcaatcgtccgatccggagccgggactgtcgggcgtacacaaatcgcccgcagaagcg
cggccgtctggaccgatggctgtgtagaagtactcgccgatagtggaaaccgacgccccagcactcgtccgagggcaaag
aaatagagtagatgccgaccggatctgtcgatcgacaagctcgagtttctccataataatgtgtgagtagttcccagata
agggaattagggttcctatagggtttcgctcatgtgttgagcatataagaaacccttagtatgtatttgtatttgtaaaa
tacttctatcaataaaatttctaattcctaaaaccaaaatccagtactaaaatccagatcccccgaattaattcggcgtt
aattcagtacattaaaaacgtccgcaatgtgttattaagttgtctaagcgtcaatttgtttacaccacaatatatcctgc
caccagccagccaacagctccccgaccggcagctcggcacaaaatcaccactcgatacaggcagcccatcagtccgggac
ggcgtcagcgggagagccgttgtaaggcggcagactttgctcatgttaccgatgctattcggaagaacggcaactaagct
gccgggtttgaaacacggatgatctcgcggagggtagcatgttgattgtaacgatgacagagcgttgctgcctgtgatca
ccgcggtttcaaaatcggctccgtcgatactatgttatacgccaactttgaaaacaactttgaaaaagctgttttctggt
atttaaggttttagaatgcaaggaacagtgaattggagttcgtcttgttataattagcttcttggggtatctttaaatac
tgtagaaaagaggaaggaaataataaatggctaaaatgagaatatcaccggaattgaaaaaactgatcgaaaaataccgc
tgcgtaaaagatacggaaggaatgtctcctgctaaggtatataagctggtgggagaaaatgaaaacctatatttaaaaat
gacggacagccggtataaagggaccacctatgatgtggaacgggaaaaggacatgatgctatggctggaaggaaagctgc
ctgttccaaaggtcctgcactttgaacggcatgatggctggagcaatctgctcatgagtgaggccgatggcgtcctttgc
tcggaagagtatgaagatgaacaaagccctgaaaagattatcgagctgtatgcggagtgcatcaggctctttcactccat
cgacatatcggattgtccctatacgaatagcttagacagccgcttagccgaattggattacttactgaataacgatctgg
ccgatgtggattgcgaaaactgggaagaagacactccatttaaagatccgcgcgagctgtatgatttttttaaagacggaa
aagcccgaagaggaacttgtcttttcccacggcgacctgggagacagcaacatctttgtgaaagatggcaaagtaagtgg
ctttattgatcttgggagaagcggcagggcggacaagtggtatgacattgccttctgcgtccggtcgatcagggaggata
tcggggaagaacagtatgtcgagctatttttgacttactggggatcaagcctgattgggagaaaataaaatattatatt
ttactggatgaattgttttagtacctagaatgcatgaccaaaatcccttaacgtgagttttcgttccactgagcgtcaga
ccccgtagaaaagatcaaaggatcttcttgagatcctttttttctgcgcgtaatctgctgcttgcaaacaaaaaaaccac
cgctaccagcggtggtttgtttgccggatcaagagctaccaactctttttccgaaggtaactggcttcagcagagcgcag
ataccaaatactgtccttctagtgtagccgtagttaggccaccacttcaagaactctgtagcaccgcctacatacctcgc
tctgctaatcctgttaccagtggctgctgccagtggcgataagtcgtgtcttaccgggttggactcaagacgatagttac
cggataaggcgcagcggtcgggctgaacggggggttcgtgcacacagcccagcttggagcgaacgacctacaccgaactg
agatacctacagcgtgagctatgagaaagcgccacgcttcccgaagggagaaaggcggacaggtatccggtaagcggcag
ggtcggaacaggagagcgcacgagggagcttccagggggaaacgcctggtatctttatagtcctgtcgggtttcgccacc

tctgacttgagcgtcgatttttgtgatgctcgtcaggggggcggagcctatggaaaaacgccagcaacgcggcctttttа
cggttcctggccttttgctggccttttgctcacatgttctttcctgcgttatcccctgattctgtggataaccgtattac
cgcctttgagtgagctgataccgctcgccgcagccgaacgaccgagcgcagcgagtcagtgagcgaggaagcggaagagc
gcctgatgcggtattttctccttacgcatctgtgcggtatttcacaccgcatatggtgcactctcagtacaatctgctct
gatgccgcatagttaagccagtatacactccgctatcgctacgtgactgggtcatggctgcgccccgacacccgccaaca
cccgctgacgcgccctgacgggcttgtctgctcccggcatccgcttacagacaagctgtgaccgtctccgggagctgcat
gtgtcagaggttttcaccgtcatcaccgaaacgcgcgaggcagggtgccttgatgtgggcgccggcggtcgagtggcgac
ggcgcggcttgtccgcgccctggtagattgcctggccgtaggccagccatttttgagcggccagcggccgcgataggccg
acgcgaagcggcggggcgtagggagcgcagcgaccgaagggtaggcgctttttgcagctcttcggctgtgcgctggccag
acagttatgcacaggccaggcgggttttaagagttttaataagttttaaagagttttaggcggaaaaatcgcctttttc
tcttttatatcagtcacttacatgtgtgaccggttcccaatgtacggctttgggttcccaatgtacgggttccggttccc
aatgtacggctttgggttcccaatgtacgtgctatccacaggaaagagaccttttcgacctttttcccctgctagggcaa
tttgccctagcatctgctccgtacattaggaaccggcggatgcttcgccctcgatcaggttgcggtagcgcatgactagg
atcgggccagcctgccccgcctcctccttcaaatcgtactccggcaggtcatttgacccgatcagcttgcgcacggtgaa
acagaacttcttgaactctccggcgctgccactgcgttcgtagatcgtcttgaacaaccatctggcttctgccttgcctg
cggcgcggcgtgccaggcggtagagaaaacggccgatgccgggatcgatcaaaaagtaatcggggtgaaccgtcagcacg
tccgggttcttgccttctgtgatctcgcggtacatccaatcagctagctcgatctcgatgtactccggccgcccggtttc
gctctttacgatcttgtagcggctaatcaaggcttcaccctcggataccgtcaccaggcggccgttcttggccttcttcg
tacgctgcatggcaacgtgcgtggtgtttaaccgaatgcaggtttctaccaggtcgtctttctgctttccgccatcggct
cgccggcagaacttgagtacgtccgcaacgtgtggacggaacacgcggccgggcttgtctcccttcccttcccggtatcg
gttcatggattcggttagatgggaaaccgccatcagtaccaggtcgtaatcccacacactggccatgccggccggccctg
cggaaacctctacgtgcccgtctggaagctcgtagcggatcacctcgccagctcgtcggtcacgcttcgacagacggaaa
acggccacgtccatgatgctgcgactatcgcgggtgcccacgtcatagagcatcggaacgaaaaaatctggttgctcgtc
gcccttgggcggcttcctaatcgacggcgcaccggctgccggcggttgccgggattctttgcggattcgatcagcggccg
cttgccacgattcaccggggcgtgcttctgcctcgatgcgttgccgctgggcggcctgcgcggccttcaacttctccacc
aggtcatcacccagcgccgcgccgatttgtaccgggccggatggtttgcgaccgtcacgccgattcctcgggcttggggg
ttccagtgccattgcagggccggcagacaacccagccgcttacgcctggccaaccgcccgttcctccacacatggggcat
tccacggcgtcggtgcctggttgttcttgattttccatgccgcctcctttagccgctaaaattcatctactcatttattc
atttgctcatttactctggtagctgcgcgatgtattcagatagcagctcggtaatggtcttgccttggcgtaccgcgtac
atcttcagcttggtgtgatcctccgccggcaactgaaagttgacccgcttcatggctggcgtgtctgccaggctggccaa
cgttgcagccttgctgctgcgtgcgctcggacggccggcacttagcgtgtttgtgcttttgctcattttctctttacctc
attaactcaaatgagttttgatttaatttcagcggccagcgcctggacctcgcgggcagcgtcgccctcgggttctgatt
caagaacggttgtgccggcggcggcagtgcctgggtagctcacgcgctgcgtgatacgggactcaagaatgggcagctcg
tacccggccagcgcctcggcaacctcaccgccgatgcgcgtgcctttgatcgcccgcgacacgacaaaggccgcttgtag
ccttccatccgtgacctcaatgcgctgcttaaccagctccaccaggtcggcggtggcccatatgtcgtaagggcttggct
gcaccggaatcagcacgaagtcggctgccttgatcgcggacacagccaagtccgccgcctggggcgctccgtcgatcact
acgaagtcgcgccggccgatggccttcacgtcgcggtcaatcgtcgggcggtcgatgccgacaacggttagcggttgatc
ttcccgcacggccgcccaatcgcgggcactgccctggggatcggaatcgactaacagaacatcggccccggcgagttgca
gggcgcgggctagatgggttgcgatggtcgtcttgcctgacccgcctttctggttaagtacagcgataaccttcatgcgt
tccccttgcgtatttgtttatttactcatcgcatcatatacgcagcgaccgcatgacgcaagctgttttactcaaataca
catcacctttttagacggcggcgctcggtttcttcagcggccaagctggccggccaggccgccagcttggcatcagacaa
accggccaggatttcatgcagccgcacggttgagacgtgcgcgggcggctcgaacacgtacccggccgcgatcatctccg
cctcgatctcttcggtaatgaaaaacggttcgtcctggccgtcctggtgcggtttcatgcttgttcctcttggcgttcat
tctcggcggccgccagggcgtcggcctcggtcaatgcgtcctcacggaaggcaccgcgccgcctggcctcggtgggcgtc
acttcctcgctgcgctcaagtgcgcggtacagggtcgagcgatgcacgccaagcagtgcagccgcctctttcacggtgcg
gccttcctggtcgatcagctcgcgggcgtgcgcgatctgtgccggggtgagggtagggcgggggccaaacttcacgcctc
gggccttggcggcctcgcgcccgctccgggtgcggtcgatgattagggaacgctcgaactcggcaatgccggcgaacacg
gtcaacaccatgcggccggccggcgtggtggtgtcggcccacggctctgccaggctacgcaggcccgcgccggcctcctg
gatgcgctcggcaatgtccagtaggtcgcgggtgctgcgggccaggcggtctagcctggtcactgtcacaacgtcgccag
ggcgtaggtggtcaagcatcctggccagctccgggcggtcgcgcctggtgccggtgatcttctcggaaaacagcttggtg
cagccggccgcgtgcagttcggcccgttggttggtcaagtcctggtcgtcggtgctgacgcgggcatagcccagcaggcc
agcggcggcgctcttgttcatggcgtaatgtctccggttctagtcgcaagtattctactttatgcgactaaaacacgcga

caagaaaacgccaggaaaagggcagggcggcagcctgtcgcgtaacttaggacttgtgcgacatgtcgttttcagaagac
ggctgcactgaacgtcagaagccgactgcactatagcagcggaggggttggatcaaagtactttgatcccgaggggaacc
ctgtggttggcatgcacatacaaatggacgaacggataaaccttttcacgcccttttaaatatccgttattctaataaac
gctcttttctcttaggtttacccgccaatatatcctgtcaaacactgatagtttaaactgaaggcgggaaacgacaatct
gatccaagctcaagctgctctagcattcgccattcaggctgcgcaactgttgggaagggcgatcggtgcgggcctcttcg
ctattacgccagctggcgaaagggggatgtgctgcaaggcgattaagttgggtaacgccagggttttcccagtcacgacg
ttgtaaaacgacggccagtgccaAGCTTGCCACCGGTCAACATGTGGAGCACGACACACTTGTCTAC
TCCAAAAATATCA
AAGATACAGTCTCAGAAGACCAAAGGGCAATTGAGACTTTTCAACAAAGGGTAATATC
CGGAAACCTCCTCGGATTCCAT
TGCCCAGCTATCTGTCACTTTATTGTGAAGATAGTGGAAAAGGAAGGTGGCTCCTACA
AATGCCATCATTGCGATAAAGG
AAAGGCCATCGTTGAAGATGCCTCTGCCGACAGTGGTCCCAAAGATGGACCCCCACCC
ACGAGGAGCATCGTGGAAAAAG
AAGACGTTCCAACCACGTCTTCAAAGCAAGTGGATTGATGTGATAACATGGTGGAGCA
CGACACACTTGTCTACTCCAAA
AATATCAAAGATACAGTCTCAGAAGACCAAAGGGCAATTGAGACTTTTCAACAAAGGG
TAATATCCGGAAACCTCCTCGG
ATTCCATTGCCCAGCTATCTGTCACTTTATTGTGAAGATAGTGGAAAAGGAAGGTGGCT
CCTACAAATGCCATCATTGCG
ATAAAGGAAAGGCCATCGTTGAAGATGCCTCTGCCGACAGTGGTCCCAAAGATGGACC
CCCACCCACGAGGAGCATCGTG
GAAAAAGAAGACGTTCCAACCACGTCTTCAAAGCAAGTGGATTGATGTGATATCTCCA
CTGACGTAAGGGATGACGCACA
ATCCCACTATCCTTCGCAAGACCCTTCCTCTATATAAGGAAGTTCATTTCATTTGGAGA
GGACGTCGAGAGTTCTCAACA
CAACATATACAAAACAAACGAATCTCAAGCAATCAAGCATTCTACTTCTATTGCAGCA
ATTTAAATCATTTCTTTTAAAG
CAAAAGCAATTTTCTGAAAATTTTCACCATTTACGAACGATAGAGATCTGGACACAAG
TTTGTACAAAAAAGCAGGCTCC
GCGGCCGCCCCCACTAGTGCCGCGCGGCAGCAAGGGTGGGCGCGCCGACCCAGCTTTC
TTGTACAAAGTGGTGATAGGGC
CCGGGATCCTGATGGTGAGCAAGGGCGAGGAGCTGTTCACCGGGGTGGTGCCCATCCT
GGTCGAGCTGGACGGCGACGTA
AACGGCCACAAGTTCAGCGTGTCCGGCGAGGGCGAGGGCGATGCCACCTACGGCAAG
CTGACCCTGAAGTTCATCTGCAC
CACCGGCAAGCTGCCCGTGCCCTGGCCCACCCTCGTGACCACCCTGACCTACGGCGTG
CAGTGCTTCAGCCGCTACCCCG
ACCACATGAAGCAGCACGACTTCTTCAAGTCCGCCATGCCCGAAGGCTACGTCCAGGA
GCGCACCATCTTCTTCAAGGAC
GACGGCAACTACAAGACCCGCGCCGAGGTGAAGTTCGAGGGCGACACCCTGGTGAAC
CGCATCGAGCTGAAGGGCATCGA
CTTCAAGGAGGACGGCAACATCCTGGGGCACAAGCTGGAGTACAACTACAACAGCCA
CAACGTCTATATCATGGCCGACA
AGCAGAAGAACGGCATCAAGGTGAACTTCAAGATCCGCCACAACATCGAGGACGGCA
GCGTGCAGCTCGCCGACCACTAC
CAGCAGAACACCCCCATCGGCGACGGCCCCGTGCTGCTGCCCGACAACCACTACCTGA
GCACCCAGTCCGCCCTGAGCAA
AGACCCCAACGAGAAGCGCGATCACATGGTCCTGCTGGAGTTCGTGACCGCCGCCGGG
ATCACTCTCGGCATGGACGAGC
TGTACAAGTGAAGATCCACCTGATCTAGAGTCCGCAAAAATCACCAGTCTCTCTCTACA
AATCTATCTCTCTCTATTTTT

CTCCAGAATAATGTGTGAGTAGTTCCCAGATAAGGGAATTAGGGTTCTTATAGGGTTTC
GCTCATGTGTTGAGCATATAA
GAAACCCTTAGTATGTATTTGTATTTGTAAAATACTTCTATCAATAAAATTTCTAATTCC
TAAAACCAAAATCCAGTGAC
Gaatt

Note : The Spe1 site (Marked by green color) is used for insertion of CDS (lack stop codon) sequence of FaBBXs.

3. Primer sequences which are used in present study

Name	Usage	Sequence	Target Gene
FaBBX15_qPCR_F	RT-qPCR Forward Primer	GTAGTAGTCAAGAGCATAA	FaBBX 15
FaBBX15_qPCR_R	RT-qPCR Reverse Primer	CAATCGGAATCAATATCG	FaBBX 15
FaBBX19_qPCR_F	RT-qPCR Forward Primer	GCAACCAAGAATAACGATT	FaBBX 19
FaBBX19_qPCR_R	RT-qPCR Reverse Primer	CATCCGATTAGGCTTCAT	FaBBX 28
FaBBX28_qPCR_F	RT-qPCR Forward Primer	TAATGCTTCTGATCTTCGCT	FaBBX 28
FaBBX28_qPCR_R	RT-qPCR Reverse Primer	GTTAAGATCCAGTTTCCGGT	FaBBX 28
BBX28OverF	FaBBX28c1 OverExpression Vectors Construction Forward	CGGGGTACCATGAAGAACTGCGAGCTCT	FaBBX 28c1
BBX28OverR	FaBBX28c1 OverExpression Vectors Construction Reverse	CGCGGATCCTCATATCAGAGGGATACGAGGC	FaBBX 28c1
SubCellF19N	Subcellular localization Vector Construction Forward	tccgcggccgcccccactagtATGCGAACACTGTGTGACGCT	FaBBX 19a1
SubCellR19N	Subcellular localization Vector Construction Reverse	ttgctgccgcgcggcactagtGATTGGTCCTCTTTTGAAGCTTG	FaBBX 19a1
SubCellF15N	Subcellular localization Vector Construction Forward	TCCGCGGCCGCCCCCACTAGTATGATATCAAACAAGAAAG	FaBBX 15a1
SubCellR15N	Subcellular localization Vector Construction Reverse	TTGCTGCCGCGCGGCACTAGTGATTTAGTAAGCATAGGAA	FaBBX 15a1

SubCellF28N	Subcellularlar localization Vector Construction Forward	TCCGCGGCCGCCCCCACTAGTATGAAGAACTGCGAGC	FaBBX28c1
SubCellR28N	Subcellular localization Vector Construction Reverse	TTGCTGCCGCGCGGCACTAGTGATATCAGAGGGATACGAGG	FaBBX28c1
19YeasF	Yeast Transactivity Analysis Vector Construction Forward	TGGCCATGGAGGCCGAATTCATGCGAACACTGTGTGACGC	FaBBX19a1
19YeasR	Yeast Transactivity Analysis Vector Construction Reverse	GCGGCCGCTGCAGGTCGACGTTGGTCCTCTTTTGAAGCTT	FaBBX19a1
28YeasF	Yeast Transactivity Analysis Vector Construction Forward	ATGGCCATGGAGGCCGAATTCATGAAGAACTGCGAGCTCT	FaBBX28c1
28YeasR	Yeast Transactivity Analysis Vector Construction Reverse	GCGGCCGCTGCAGGTCGACGTATCAGAGGGATACGAGGCT	FaBBX28c1
pBBBX15F	Yeast Transactivity Analysis Vector Construction Forward	GGAATTCATGATATCAAACAAGAAAGCAT	FaBBX15a1
pBBBX15R	Yeast Transactivity Analysis Vector Construction Reverse	GCGTCGACGTTTAGTAAGCATAGGAAATGT	FaBBX15a1
proFaBBX28c1_GUS_F	Promoter::GUS Vector Construction Foward	TATGACATGATTACGAATTCGATTATCTTCCTAGGGCTTGTAATCC	proFaBBX28c1
proFaBBX28c1_GUS_R	Promoter::GUS Vector Construction Reverse	taccctcagatcTACCATGGCTCCGGTGAGAGGACTCAGA	proFaBBX28c1
FT-F	RT-qPCR Forward Primer	CTGGAACAACCTTTGGCAAT	AtFT
FT-R	RT-qPCR Reverse Primer	AGCCACTCTCCCTCTGACAA	AtFT
CO-F	RT-qPCR Forward Primer	ATTCTGCAAACCCACTTGCT	AtCO
CO-R	RT-qPCR Reverse Primer	CCTCCTTGGCATCCTTATCA	AtCO
SOC1-F	RT-qPCR Forward Primer	AATTCGCCAGCTCCAATATG	AtSOC1
SOC1-R	RT-qPCR Reverse Primer	CCTCGATTGAGCATGTTCCTA	AtSOC1
AtActin2-F	RT-qPCR Forward Primer	GACCTTTAACTCTCCCGCTATG	AtActin2
AtActin2-R	RT-qPCR Reverse Primer	GAGACACACCATCACCAGAAT	AtActin2
FaACTIN2F	RT-qPCR Forward Primer	GCTAATCGTGAGAAGATGAC	FaACTIN2

FaACTIN2R	RT-qPCR Reverse Primer	GCTAATCGTGAGAAGATGAC	FaACTIN2

4. The Perl script used in present study.

```
# Perl script for prediction of protein parameters
die "perl $0 <in>  <out>" unless(@ARGV==2);
use Bio::SeqIO;
use Bio::Seq;
use Bio::Tools::SeqStats;
use Bio::Tools::pICalculator;
use Data::Dumper;

#Read in the sequences

my $in = Bio::SeqIO->new(
-file   => "$ARGV[0]",
-format => 'Fasta'
);
open OUT,">$ARGV[1]" or die "$!";
print OUT "#ID\tlength\tMV(Da)\tpI\n";
my $calc = Bio::Tools::pICalculator->new(-places => 2,-pKset => 'EMBOSS');

# predict and write out

while ( my $seq = $in->next_seq() ) {
my ( $id, $sequence, $desc ) = ( $seq->id, $seq->seq, $seq->desc );
my $weight = Bio::Tools::SeqStats ->get_mol_wt($seq);
$calc->seq($seq);
   my $iep = $calc->iep;
   print OUT sprintf("%s\t%s\t%s\t%s\n",
               $seq->id,
               $seq->length,
               "$weight->[0]",
               $iep);
}
$in->close();
close(OUT);
```
