# Supplementary material for: An Evolutionary Analysis of B-Box Transcription Factors in Strawberry Reveals the Role of FaBBx28c1 in the Regulation of Flowering Time
Source: Int J Mol Sci. 2021 Oct 29;22(21):11766. doi: 10.3390/ijms222111766 (PMC8583817; doi:10.3390/ijms222111766)
Supplement: Supplementary file 1 [file ijms-22-11766-s001.zip › SFiles/Table S8.pdf]

A statistical comparison of the *Ka/Ks* value

	Ka/Ks of *FvBBX* gene pairs	*Ka/Ks* of Genome wide gene pairs in cultivated strawberry
Ka/Ks of *FaBBX* gene pairs	4.82E-08	1.72E-04
Ka/Ks of Genome wide gene pairs in wild strawberry	4.41E-01	2.32E-05
