# Supplementary material for: An Evolutionary Analysis of B-Box Transcription Factors in Strawberry Reveals the Role of FaBBx28c1 in the Regulation of Flowering Time
Source: Int J Mol Sci. 2021 Oct 29;22(21):11766. doi: 10.3390/ijms222111766 (PMC8583817; doi:10.3390/ijms222111766)
Supplement: Supplementary file 1 [file ijms-22-11766-s001.zip › SFiles/TableS12.pdf]

TableS 12

The Blast result of four selected FaBBXs

GeneBank_ACCESSION	NCBI_Name	Gene_Name	Blast_Hit	Blast_Identity
MZ131644.1	FaBBX5	FaBBX5a1	maker-Fvb2-1-augustus-gene-264.73-mRNA-1	98.11%
MG772809.1	FaBBX15	FaBBX15a1	maker-Fvb1-1-augustus-gene-151.38-mRNA-1	99.03%
MZ131645.1	FaBBX19	FaBBX19a1	maker-Fvb1-1-augustus-gene-216.44-mRNA-1	98.72%
MN866310.1	FaBBX28	FaBBX28c1	maker-Fvb6-1-augustus-gene-58.64-mRNA-1	99.47%
