# Supplementary figures and images for: An Evolutionary Analysis of B-Box Transcription Factors in Strawberry Reveals the Role of FaBBx28c1 in the Regulation of Flowering Time
Source: Int J Mol Sci. 2021 Oct 29;22(21):11766. doi: 10.3390/ijms222111766 (PMC8583817; doi:10.3390/ijms222111766)

Physical and Chemical Parameters of BBXs in Strawberry

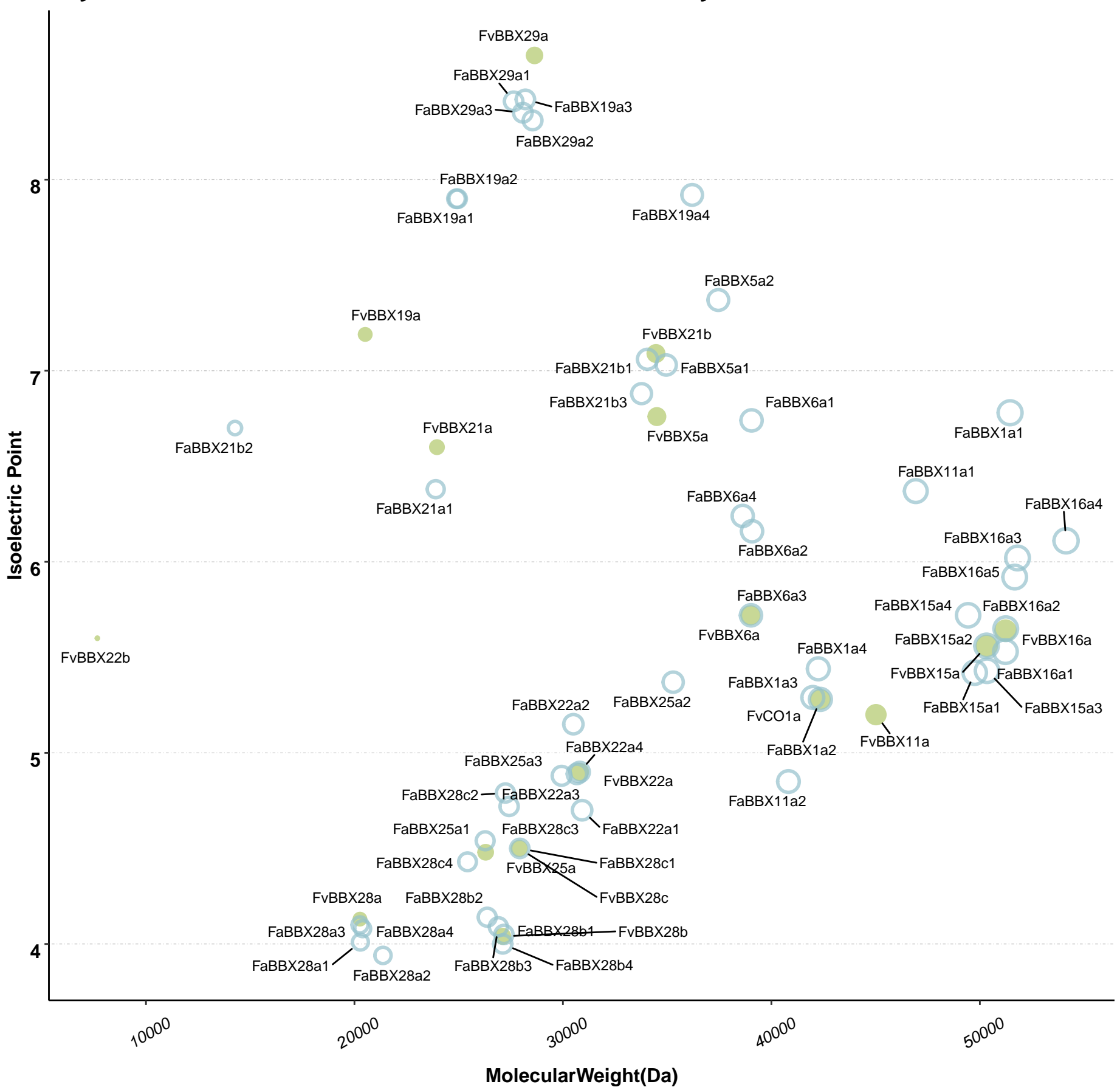

Supplement: Supplementary file 1 [file ijms-22-11766-s001.zip › SFiles/Figure S2.pdf]

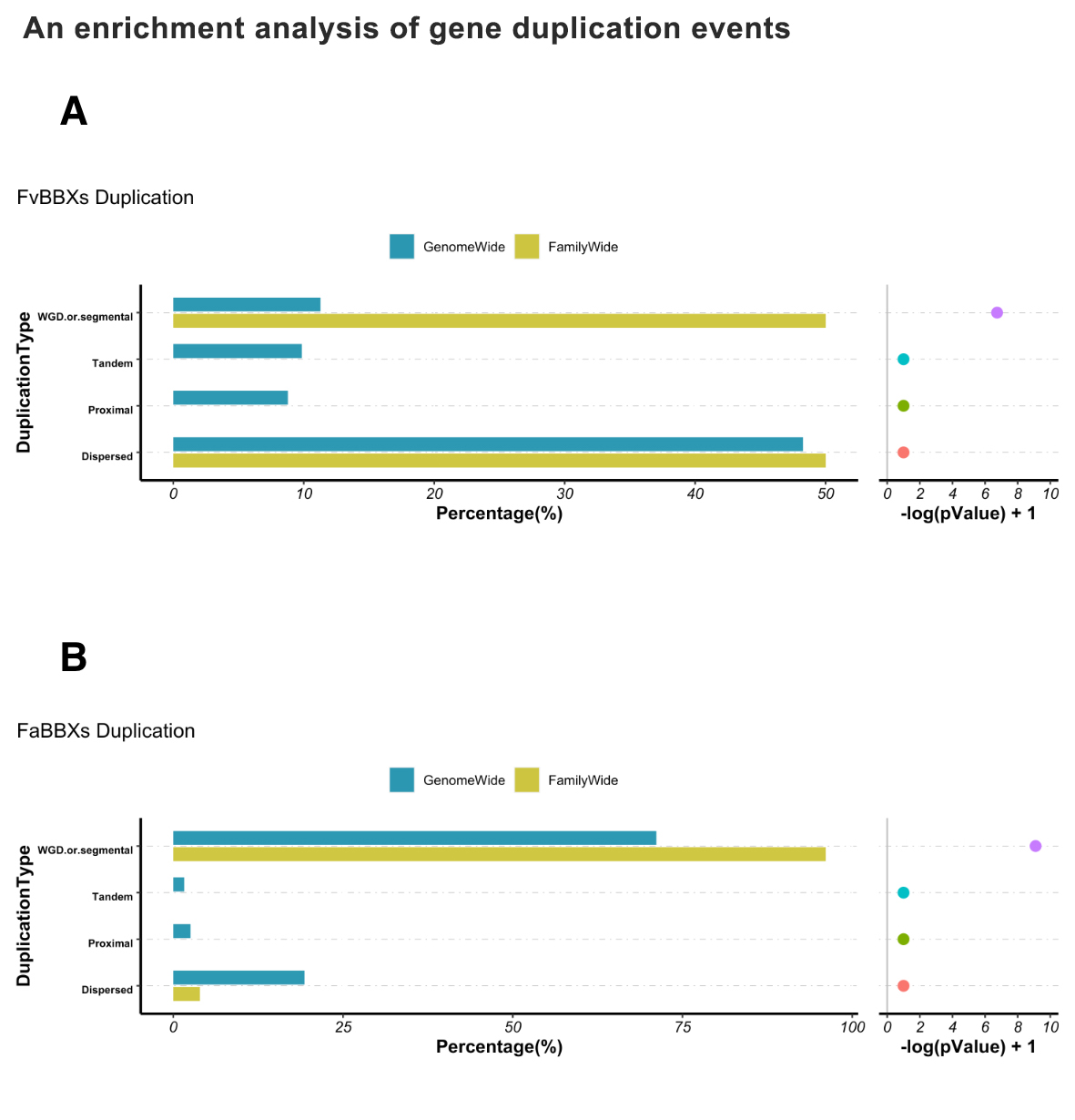

Supplement: Supplementary file 1 [file ijms-22-11766-s001.zip › SFiles/Figure S3.jpg]

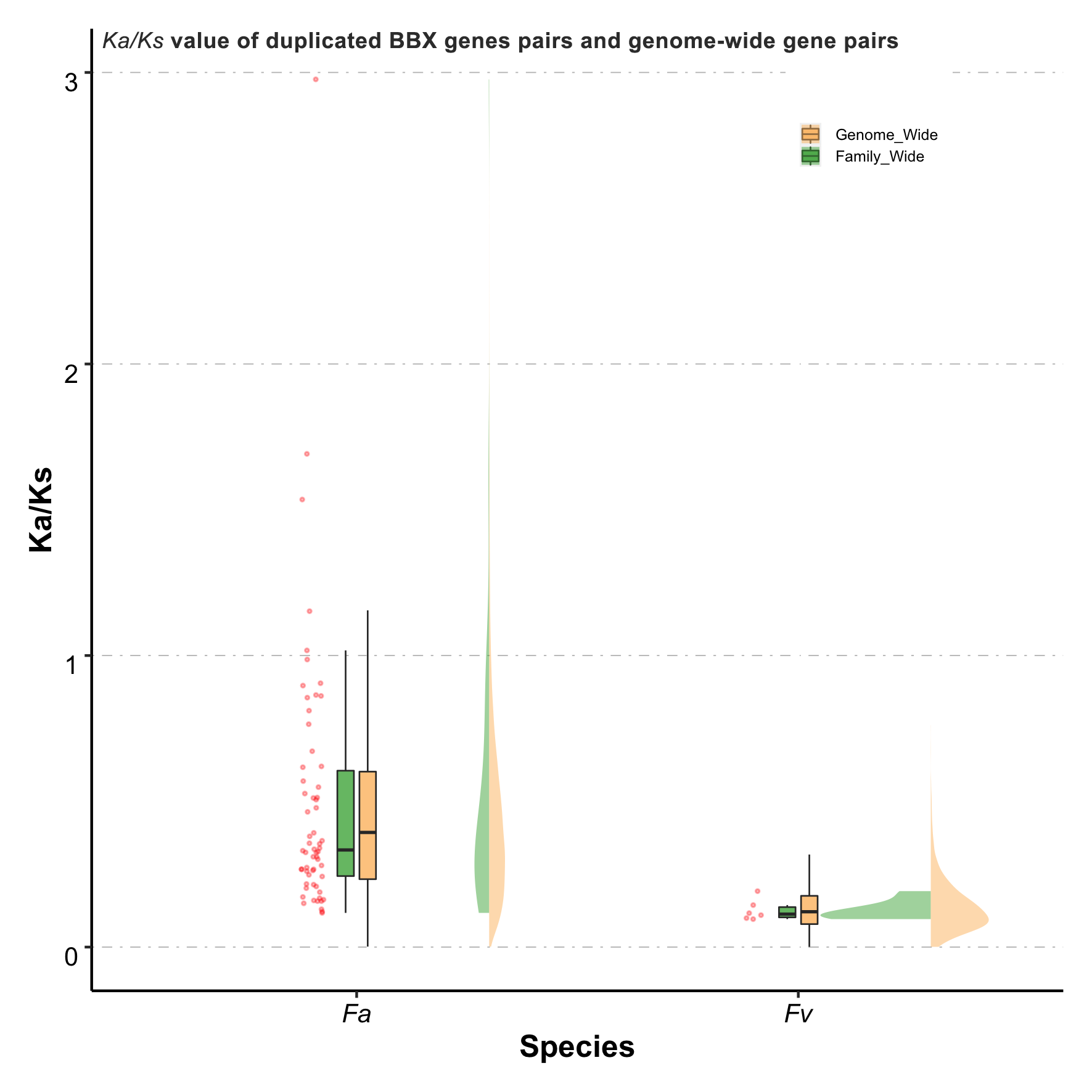

Supplement: Supplementary file 1 [file ijms-22-11766-s001.zip › SFiles/Figure S4.png]

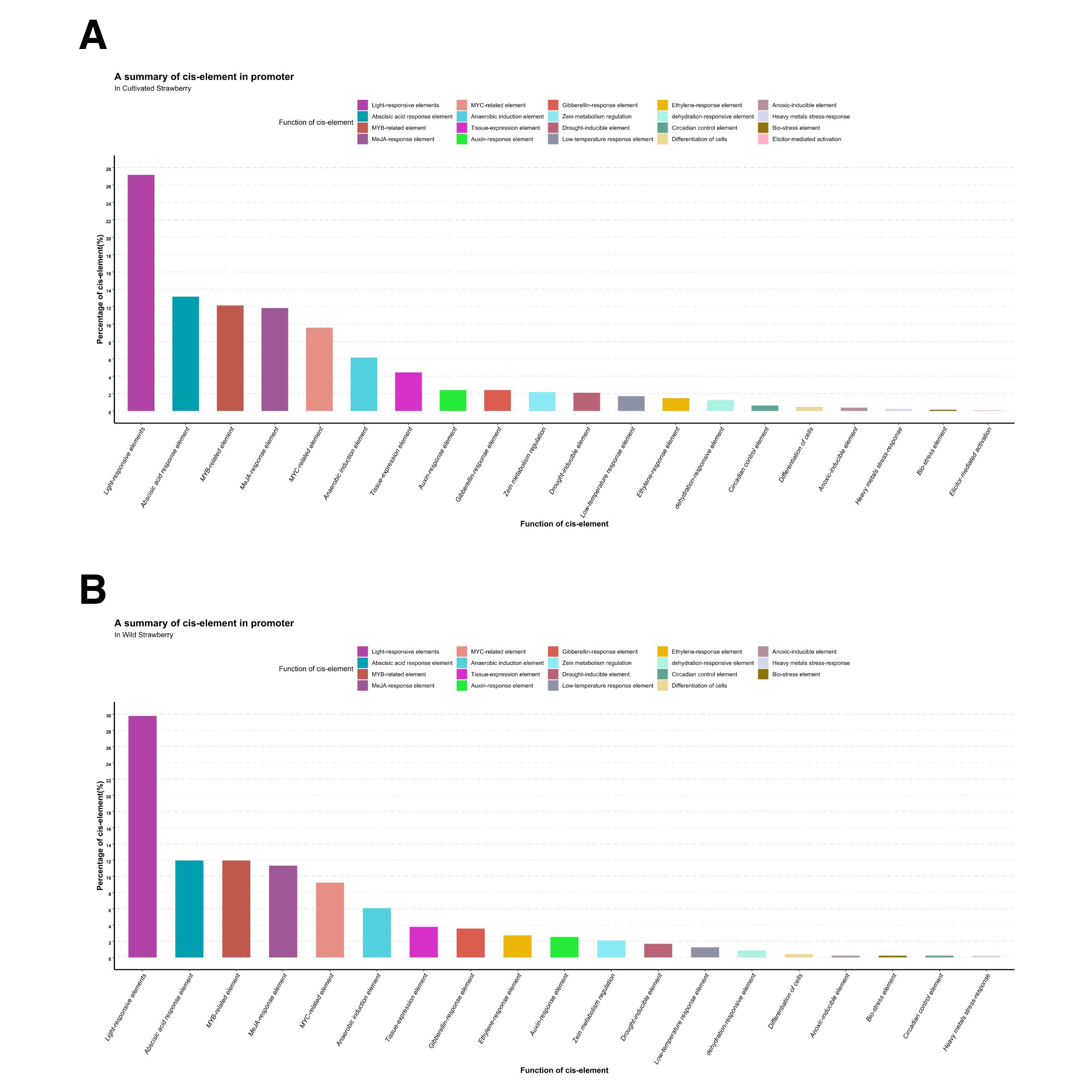

Supplement: Supplementary file 1 [file ijms-22-11766-s001.zip › SFiles/Figure S6.jpg]

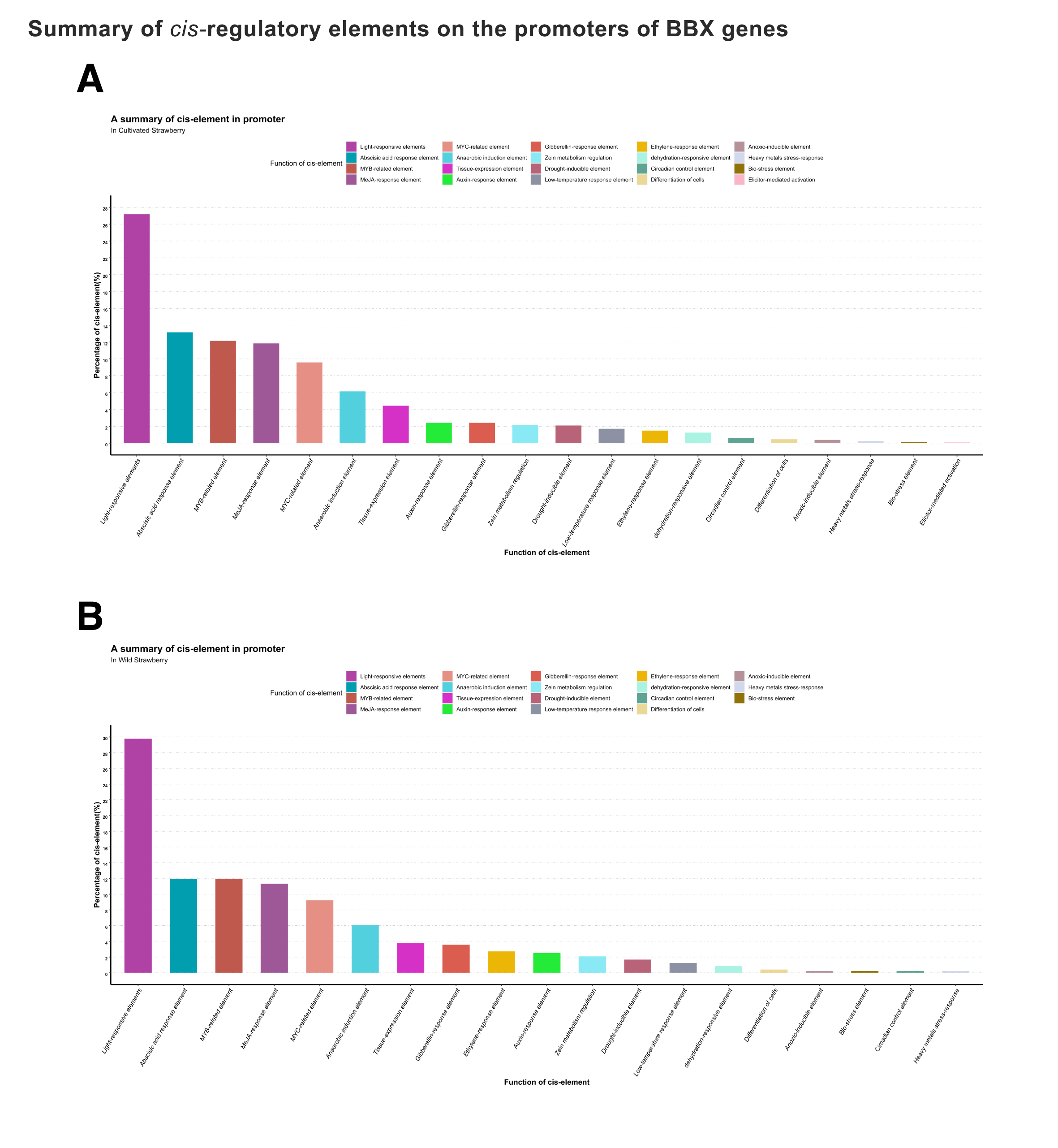

Supplement: Supplementary file 1 [file ijms-22-11766-s001.zip › SFiles/Figure S6.tif]

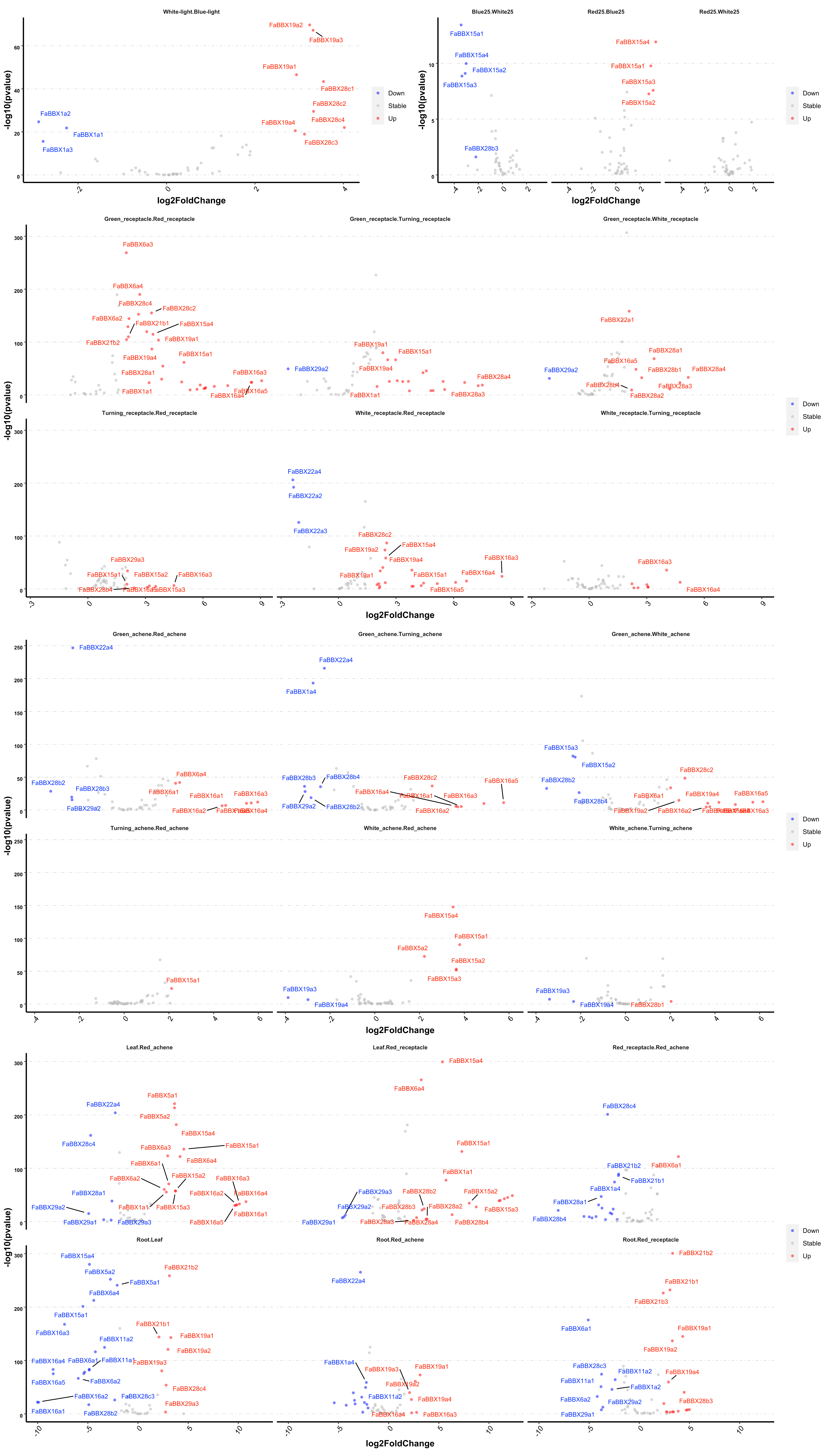

Supplement: Supplementary file 1 [file ijms-22-11766-s001.zip › SFiles/Figure S7.png]

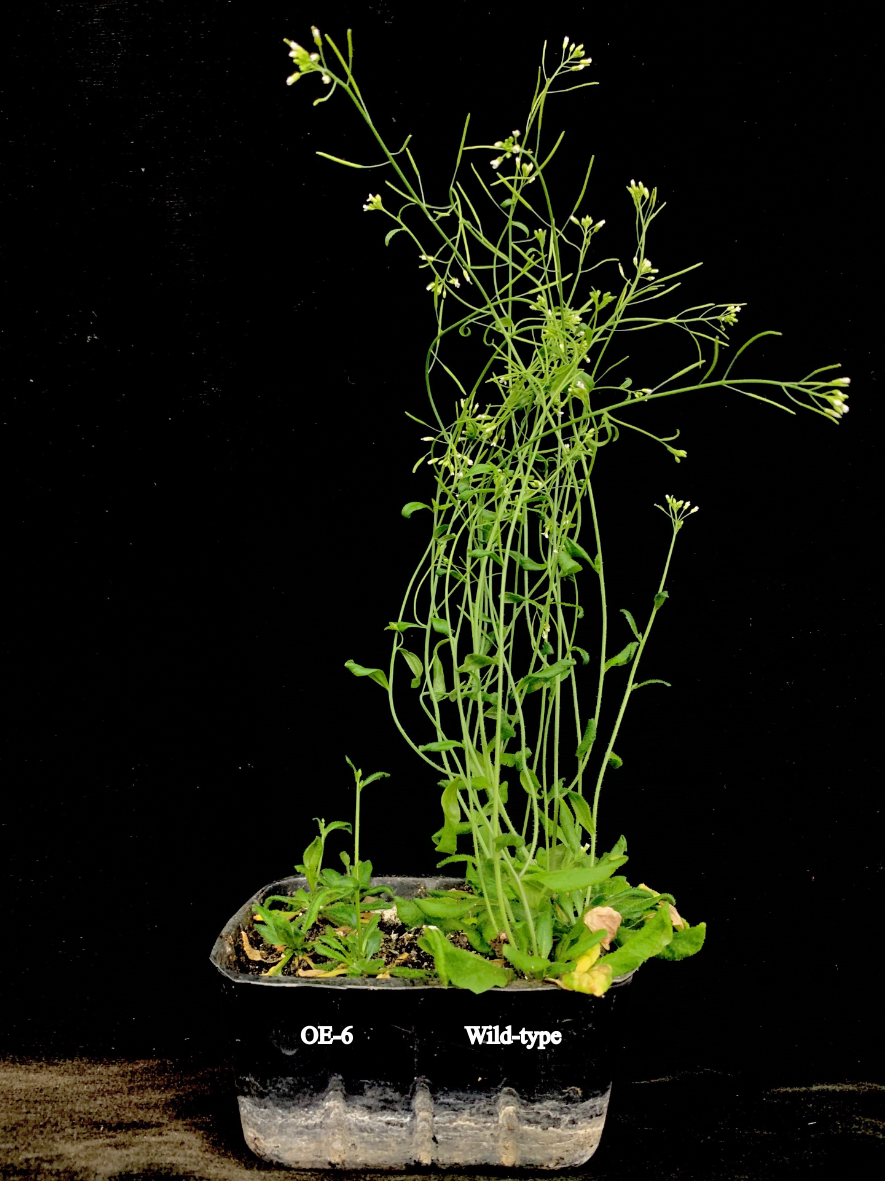

Supplement: Supplementary file 1 [file ijms-22-11766-s001.zip › SFiles/Figure S8.jpg]

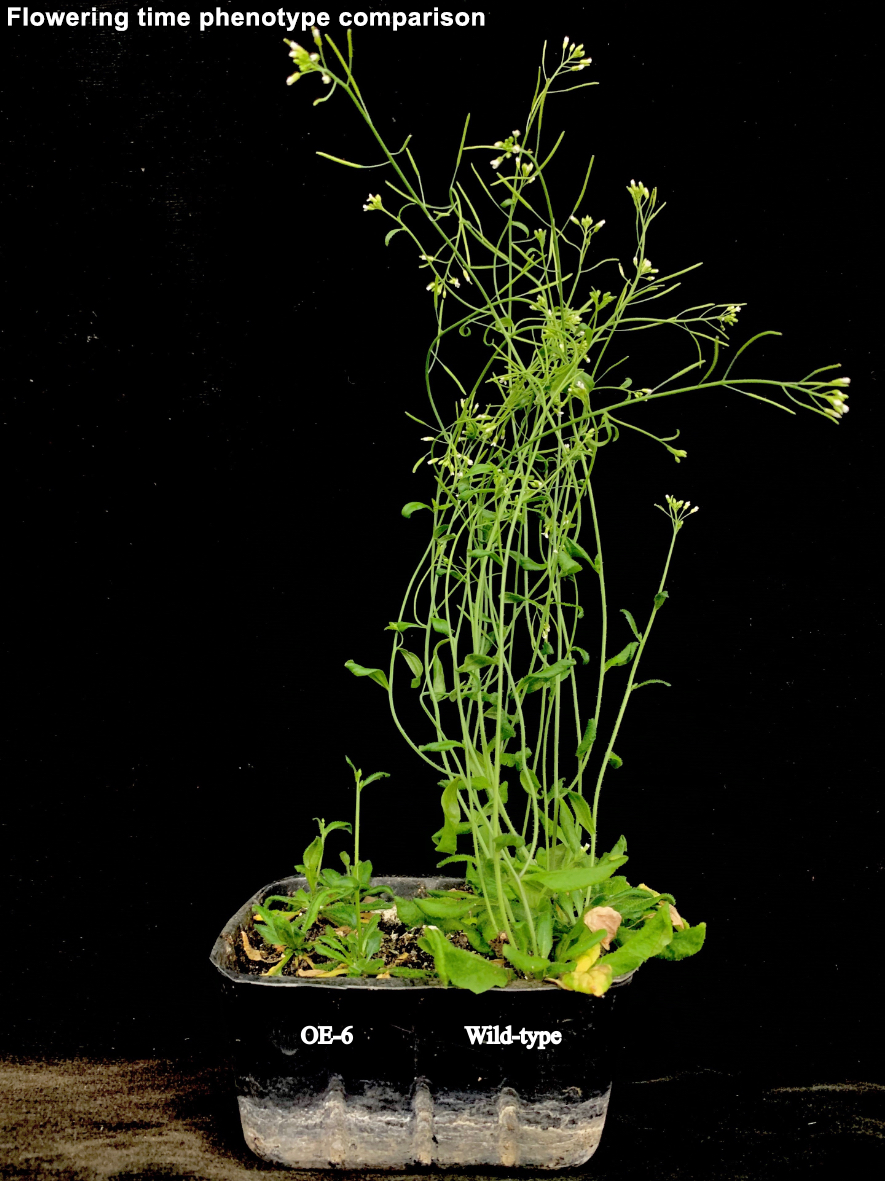

Supplement: Supplementary file 1 [file ijms-22-11766-s001.zip › SFiles/Figure S8.tif]

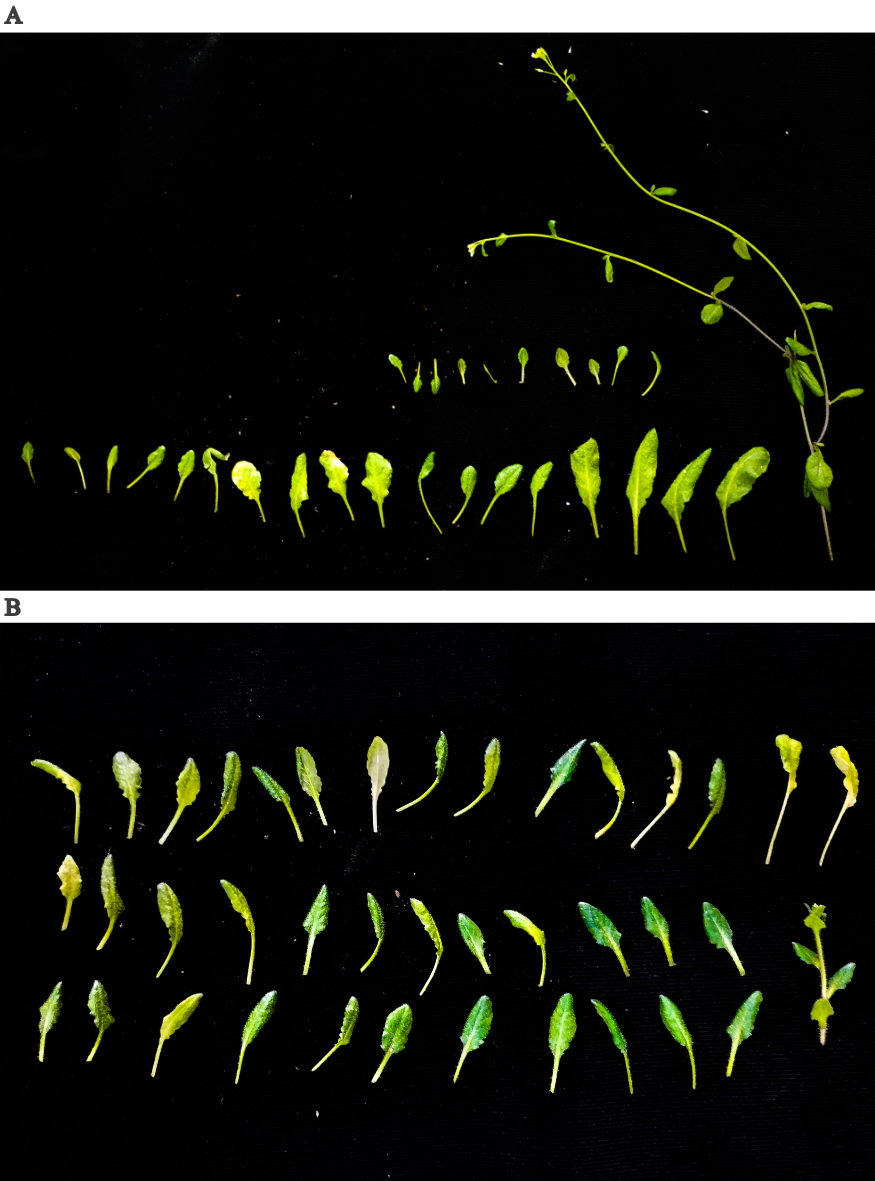

Supplement: Supplementary file 1 [file ijms-22-11766-s001.zip › SFiles/Figure S9.jpg]

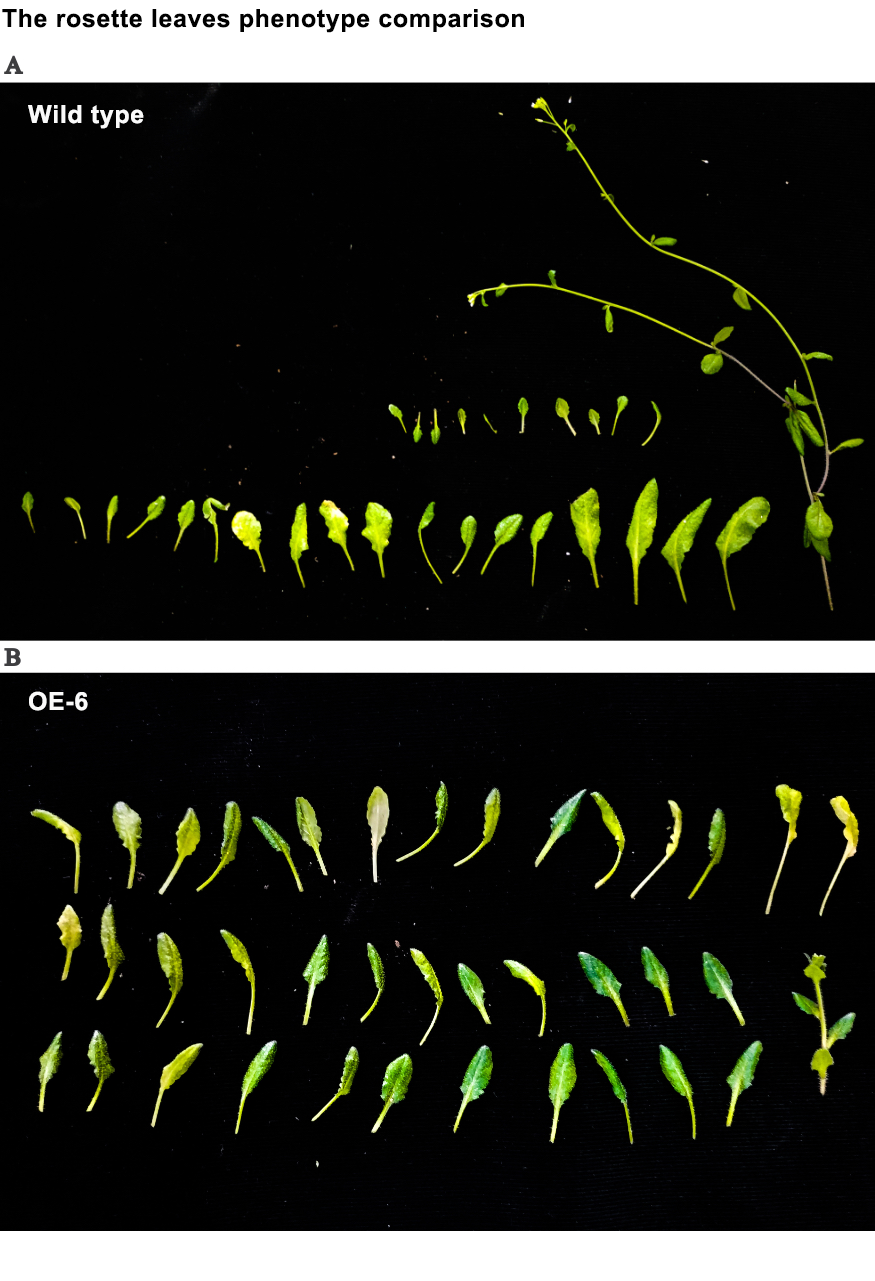

Supplement: Supplementary file 1 [file ijms-22-11766-s001.zip › SFiles/Figure S9.tif]
